# Supplementary material for: Temporal and compartment-specific signals coordinate mitotic exit with spindle position
Source: Nat Commun. 2017 Jan 24;8:14129. doi: 10.1038/ncomms14129 (PMC5286211; doi:10.1038/ncomms14129)
Supplement: Supplementary Information — Supplementary Figures, Supplementary Tables, Supplementary Methods and Supplementary References [file ncomms14129-s1.pdf]

SUPPLEMENTARY INFORMATION

Supplementary Figures (1-12)

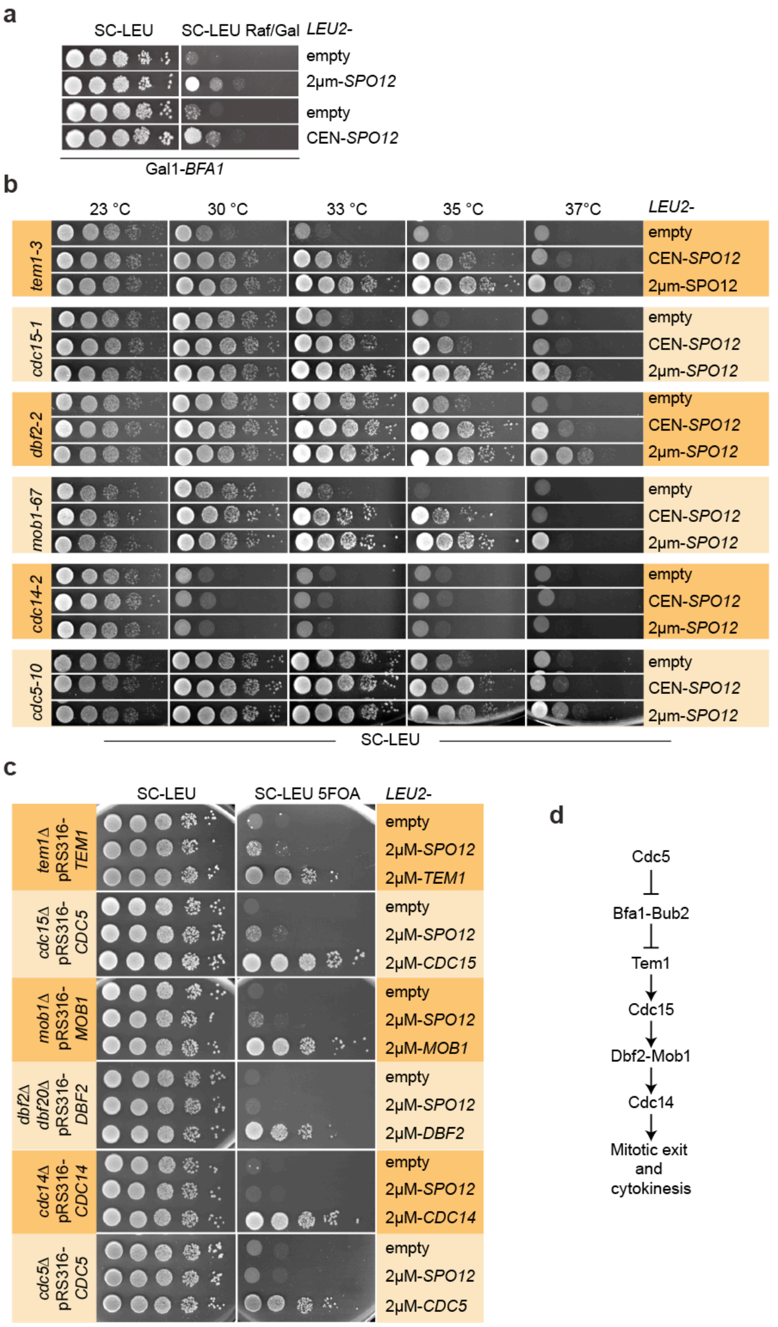

Supplementary Figure 1. Suppression of mitotic exit defects by multi-copy *SPO12*.
a-c. Effect of *SPO12* on low-copy (centromeric, pRS315) or high-copy (2 µm) plasmids to the growth of (a) Gal1-*BFA1* overexpressing cells (B) MEN temperature sensitive mutants[1-10] and (c) strains carrying gene knock-outs for MEN components. Each MEN gene knock-out was initially complemented with a *URA3*-based centromeric plasmid (pRS316) carrying the corresponding wild type gene. Serial dilutions of indicated strains were spotted on Gal1-promoter repressing (SC-LEU) or inducing (SC-LEU Raf/Gal) conditions (A) or rich plates at different temperatures (b). In c, serial dilutions were spotted on plates that allowed for

selection of *LEU2* and *URA3*-based plasmids (SC-LEU2) or *LEU2*-based plasmids only (SC-LEU 5FOA – please note that 5FOA negatively selects for *URA3*-based plasmids). In a and c, plates were incubated at 30°C.
d. Graphical representation of the MEN pathway [6,7,11-18]. Arrows are activating steps whereas capped-lines are inhibitory steps.

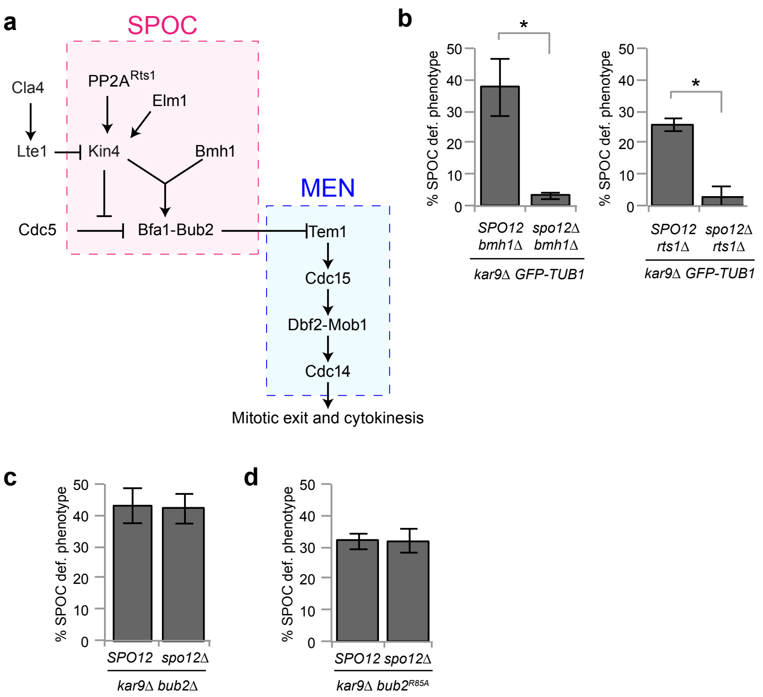

Supplementary Figure 2. Kin4 branch of the SPOC becomes dispensable for SPOC functioning in the absence of FEAR.
a. Graphical representation of the SPOC[19-29]. The arrowed lines indicate activating whereas capped lines indicate inhibitory regulations. PP2A-Rts1 dephosphorylates Kin4 to target Kin4 to the SPBs and the mother cell cortex [30]. Elm1 kinase phosphorylates a residue in the catalytic loop of Kin4 to activate it [31]. Kin4 phosphorylates Bfa1 and the 14-3-3-family-protein, Bmh1, binds to Kin4 phosphorylated Bfa1 to promote Bfa1 dissociation from SPBs [21,22,26-29]. Cdc5 phosphorylates Bfa1 to inhibit the Bfa1-Bub2 GAP activity [32,33]. Kin4 phosphorylation of Bfa1 prevents Cdc5 phosphorylation of Bfa1 [21,22,27]. Kin4 activity and localization are inhibited by Lte1 in the daughter cell [19,20].
b-d. Deletion of *SPO12* rescues SPOC deficiency of *bmh1*Δ and *rts1*Δ cells (b), but not *bub2*Δ (c) or *bub2*^{R85A} (d) cells. Each graph represents percentage of SPOC-deficient phenotypes (average of three independent experiments). Error bars show standard deviation. 100 cells are counted per cell type per experiment. Asterisks indicate significant difference according to two-tailed Student's t-test ($p<0.05$).

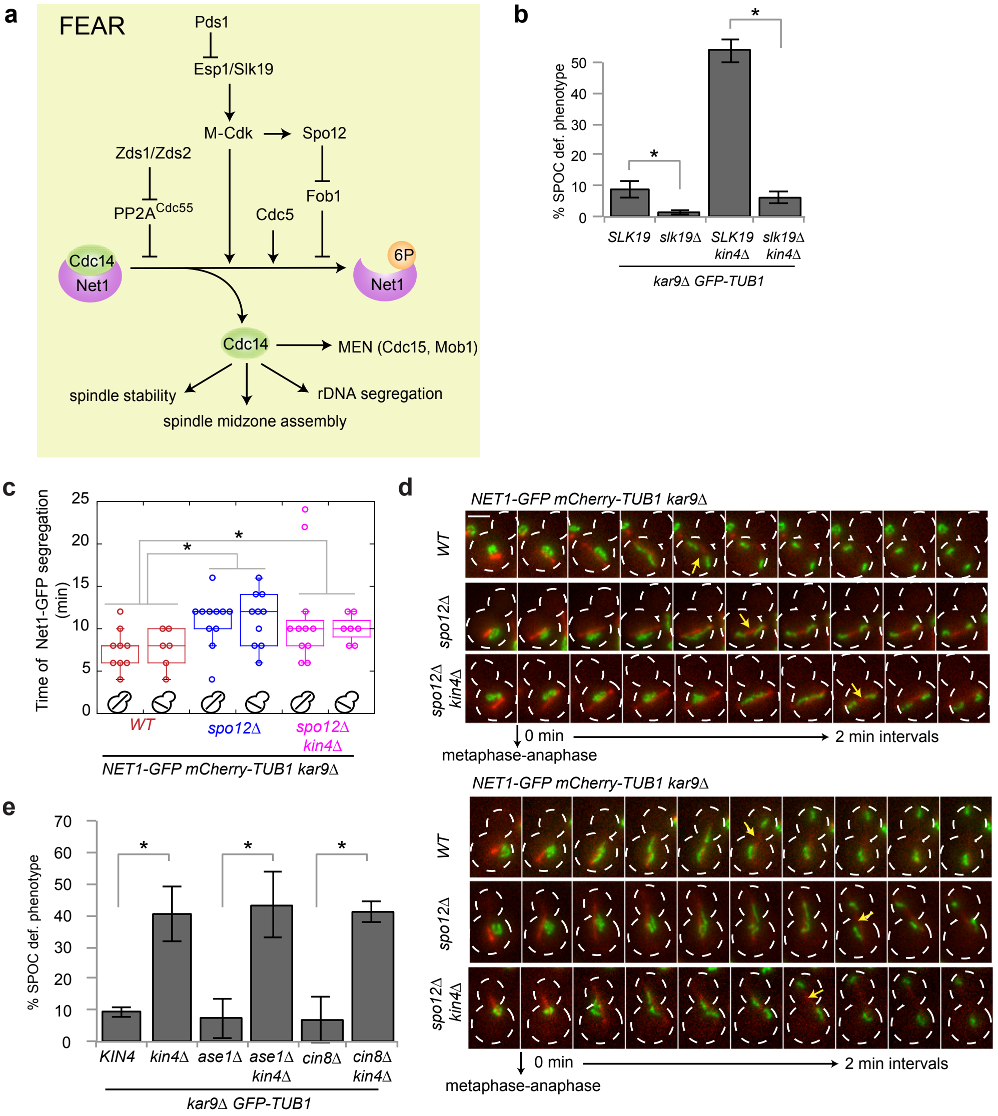

Supplementary Figure 3. rDNA segregation occurs independently of spindle orientation.

a. Graphical representation of the FEAR pathway [34-50]. The arrowed lines indicate activating whereas capped lines indicate inhibitory regulations. FEAR-network promotes transient release of Cdc14 from its nucleolar inhibitor Net1 by promoting Net1 phosphorylation by mitotic M-Cdk [38]. This requires retention of PP2A^{Cdc55} in the cytoplasm by Zds1/Zds2, separase (Esp1) activity, and Cdc5, Spo12 and Slk19 proteins [34-37,51-54]. Fob1 also retains Cdc14 in the nucleolus and this step is prevented by Spo12 [55].

b. Deletion of *SLK19* rescues SPOC deficiency of *kin4*Δ cells. Graph is average of three independent experiments. Error bars are standard deviation. 100 cells are counted per cell type per experiment.

c-d. Timing of rDNA segregation relative to metaphase-to-anaphase transition was calculated from the time-lapse movies of *NET1-GFP mCherry-TUB1 kar9*Δ cells, monitoring the rDNA binding protein Net1-GFP by time-lapse microscopy. (c) Super imposed box and whisker, and dot plots showing the timing of Net1-GFP segregation. Dots represent individual cells analyzed by time-lapse microscopy (2 min time resolution). The boxes show the lower and upper quartiles, the whiskers show the minimum and maximal values excluding outliers; outliers (shown as circles) were calculated as values greater or lower than 1.5 times the interquartile range. The lines inside the boxes are the median. Wild type cells segregated their

rDNA 8±2 min (mean±standard deviation) after metaphase-to-anaphase transition regardless of spindle orientation status. In comparison to wild type cells, the timing of rDNA segregation was 3 min delayed in *spo12*Δ and *spo12*Δ *kin4*Δ cells (11±3 min and 11±5 min respectively). Importantly timing of rDNA segregation was not affected by spindle mis-orientation in any of the cell types analyzed. Usage of a *kar9*Δ background allowed for analysis of cells during spindle mis- and normal alignment. *mCherry-TUB1* served as an internal standard for cell cycle progression and spindle orientation. (d) Representative still images from the time-lapse series of indicated *NET1-GFP mCherry-TUB1 kar9*Δ cells. Dashed lines delineate the cell boundaries. Black arrow indicates the metaphase-to-anaphase transition, whereas yellow arrow shows the time of rDNA segregation, determined by the clear separation of the two rDNA loci based on Net1-GFP. Scale bar: 3μm.
e. Effect of Ase1 and Cin8 on SPOC deficiency of *kin4*Δ cells. Graph is average of three independent experiments. Error bars show standard deviation. 100 cells were counted per strain per experiment. Asterisks indicate difference according to two-tailed Student's t-test ($p<0.05$).

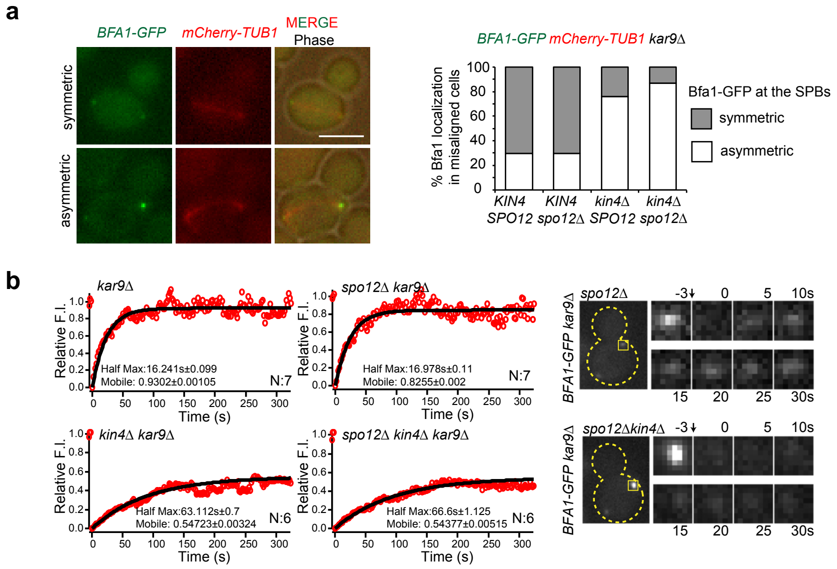

Supplementary Figure 4. Effect of FEAR on Bfa1 SPB localization.

a. Bfa1-GFP localization at SPBs during spindle misalignment. Representative images of symmetric (similar amounts at both SPBs), and asymmetric (different amounts at SPBs) Bfa1-GFP localization are shown on the left. Percentages of cells with symmetric and asymmetric Bfa1-GFP localization during spindle misalignment are shown on the right. 50 cells are counted per strain.
b. SPB binding dynamics of Bfa1-GFP determined by FRAP analysis. Average FRAP recovery curves of Bfa1-GFP in indicated cell types during spindle misalignment. Half Max: half recovery time (mean±standard error of the mean), Mobile: Mobile fraction (mean±standard error of the mean). N: number of cells analyzed. Representative images from the FRAP time-lapse series of *spo12*Δ and *SPO12* cells. Arrows indicate the time of photobleaching. Dashed lines outline cell boundaries. The area shown in the image series is indicated by the square around the Bfa1-GFP SPB signal. Scale bar: 3 μm.

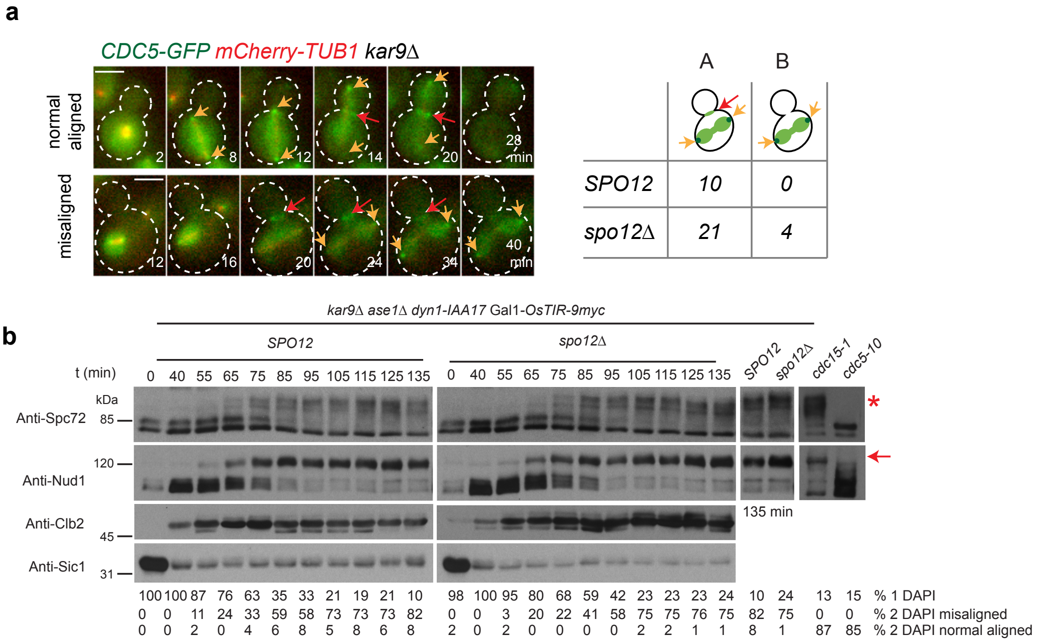

Supplementary Figure 5. Cdc5 activity during spindle misalignment.

a. Representative still images from a time-lapse series showing Cdc5-GFP localization in cells with normal (upper row) and misaligned spindle (lower row). Orange arrows indicate the SPB localization of Cdc5, whereas red arrows show the bud neck localization. Scale bar: 3µm. The localization of Cdc5-GFP was analyzed in *SPO12* and *spo12Δ* cells with misaligned spindles by live-cell imaging. Number of cells that had Cdc5-GFP in the nucleus, SPBs and bud neck (category A) as well the as number of cells that had Cdc5-GFP in the nucleus and SPBs (category B) are indicated in the table on the right.

b. Immunoblots showing Spc72 and Nud1 mobility shift during spindle misalignment. *kar9Δ ase1Δ dyn1-IAA17 Gal1-OsTIR-9myc* cells (with or without *SPO12*) were released from G1 arrest (time-point zero) under *dyn1-IAA17* depleting conditions. Samples were collected at indicated time points (minutes after G1 release). Samples of *SPO12* and *spo12Δ* cells (taken at 135 min) were loaded side-by-side for comparison of Spc72 and Nud1 mobility shift. Temperature sensitive mutants *cdc15-1* and *cdc5-10* were arrested in G1 at 23°C and released from G1 arrest at 37 °C until >90% of the cells were arrested with separated nuclei. Asterisks and arrow show the Cdc5-dependent hyperphosphorylated forms of Spc72 and Nud1 respectively. Clb2 and Sic1 served as cell cycle markers. Percentage of cells with correct (2 DAPI normal aligned) and misaligned anaphase spindles (2 DAPI misaligned) is indicated. 100 cells were counted per strain per time point.

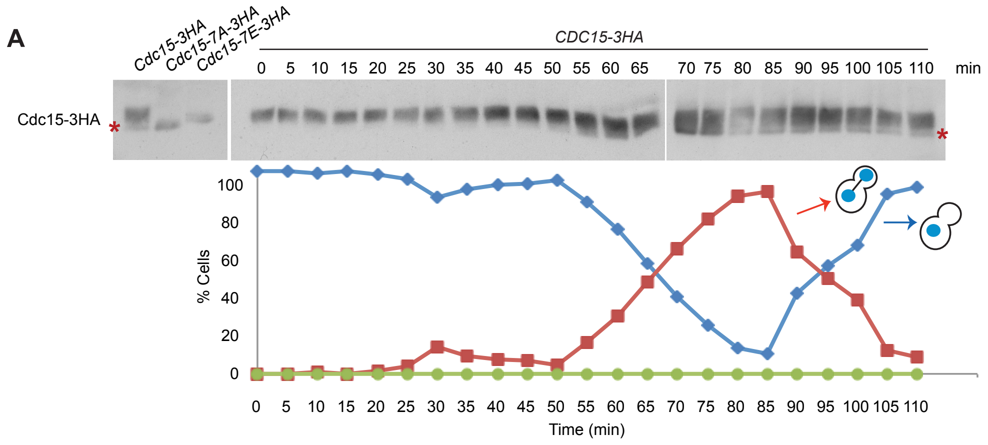

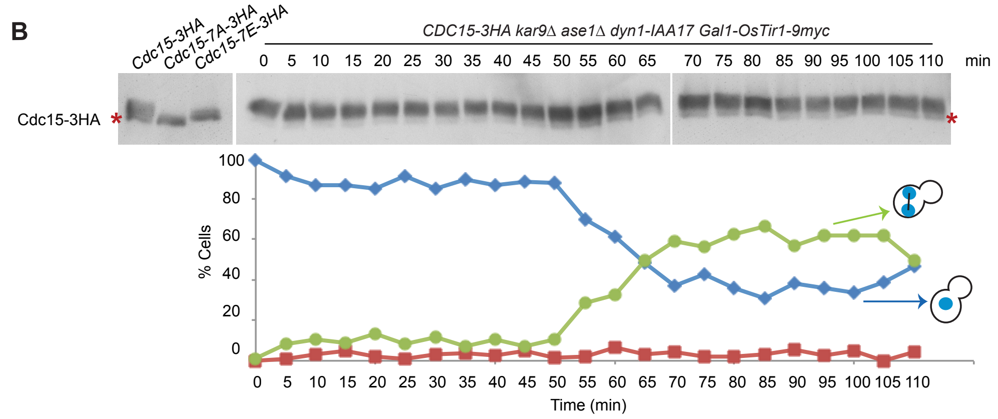

Supplementary Figure 6. Cdc15-3HA migration profile on SDS-PAGE in cells with correctly aligned and misaligned spindles.

a-b. Cells were arrested in G1 (t=0) and released from the G1 block in dynein-depleting conditions (IAA and raffinose and galactose containing medium). Samples were collected every 5 min. Asterisks indicate the dephosphorylated forms of Cdc15. Cdc15-7A and 7E mutants were included as controls. Graphs show the percentage of cells with single nucleus staining, and correctly (normal aligned, in a) or incorrectly (misaligned, in b) positioned double nuclei staining. 100 cells were counted per strain per time point.

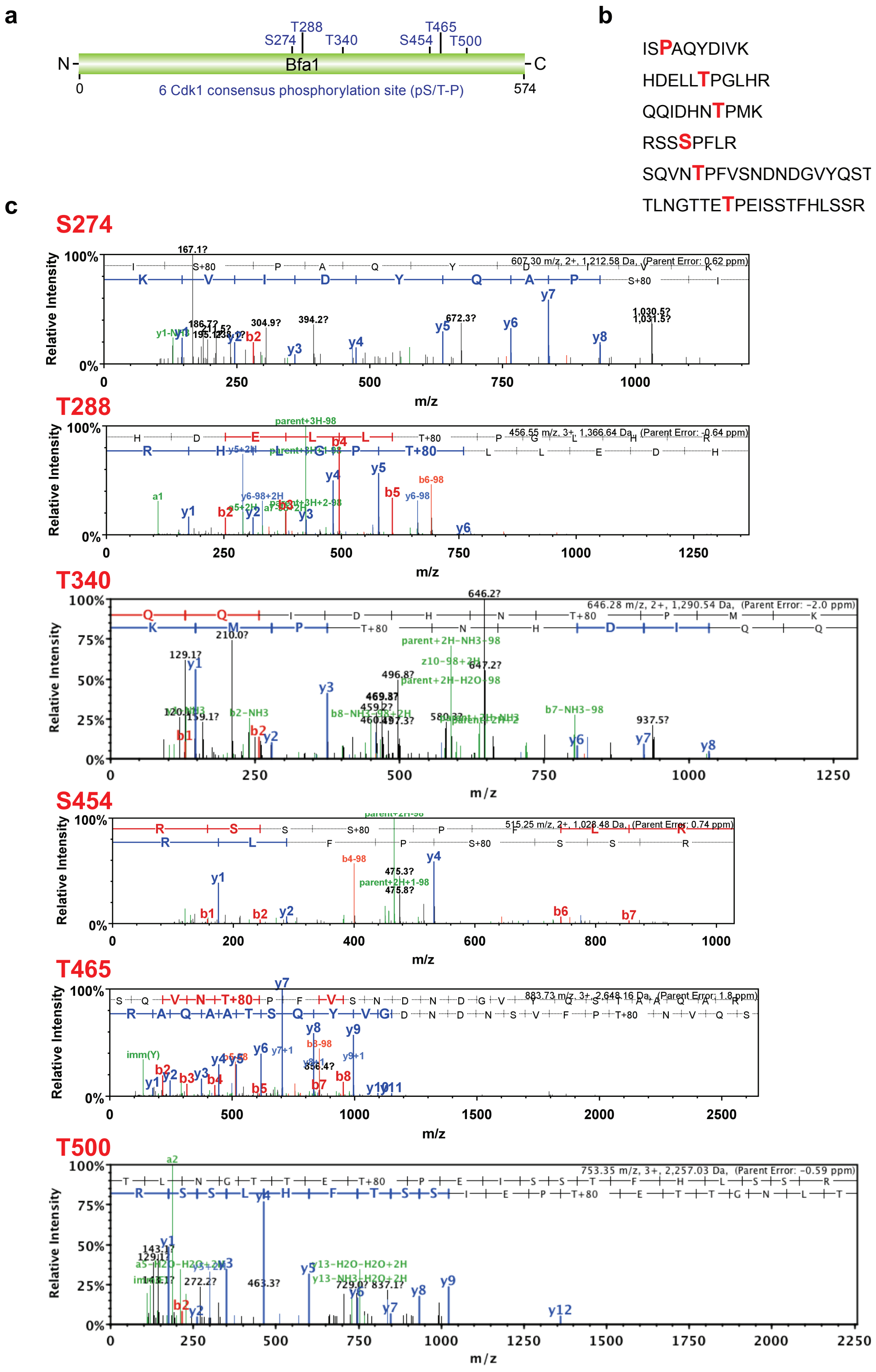

Supplementary Figure 7. In vitro Bfa1 phosphorylation by M-Cdk.

a. Schematic representation of M-Cdk minimum consensus sites (Serine or Threonine residue followed by Proline residue) on Bfa1.

b-c. M-Cdk minimum consensus sites found to be phosphorylated in vitro. **(b)** Peptide sequences. Phosphorylated residues are shown in red. **(c)** MS-MS spectra of the sequences shown in B, generated by the mass spectrometer (Scaffold 4 Proteome Software, Portland, Oregon).

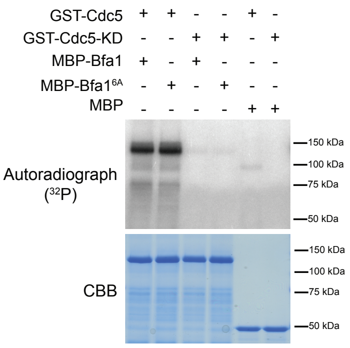

Supplementary Figure 8. In vitro phosphorylation of Bfa1 by Cdc5. Bacterially purified Bfa1 fused to Maltose binding protein (MBP-Bfa1, MBP-Bfa1-6A) was incubated with Gluthatione-S-transferase (GST) tagged Cdc5 or Cdc5-kinase dead (Cdc5-KD), purified from yeast in a kinase reaction. Reactions were performed in the presence of γ^{32}P-ATP. Autoradiograph shows incorporation of radioactive ATP into MBP-Bfa1 (on the top). Substrate levels are detected by Colloidal Coomassie staining (on the bottom).

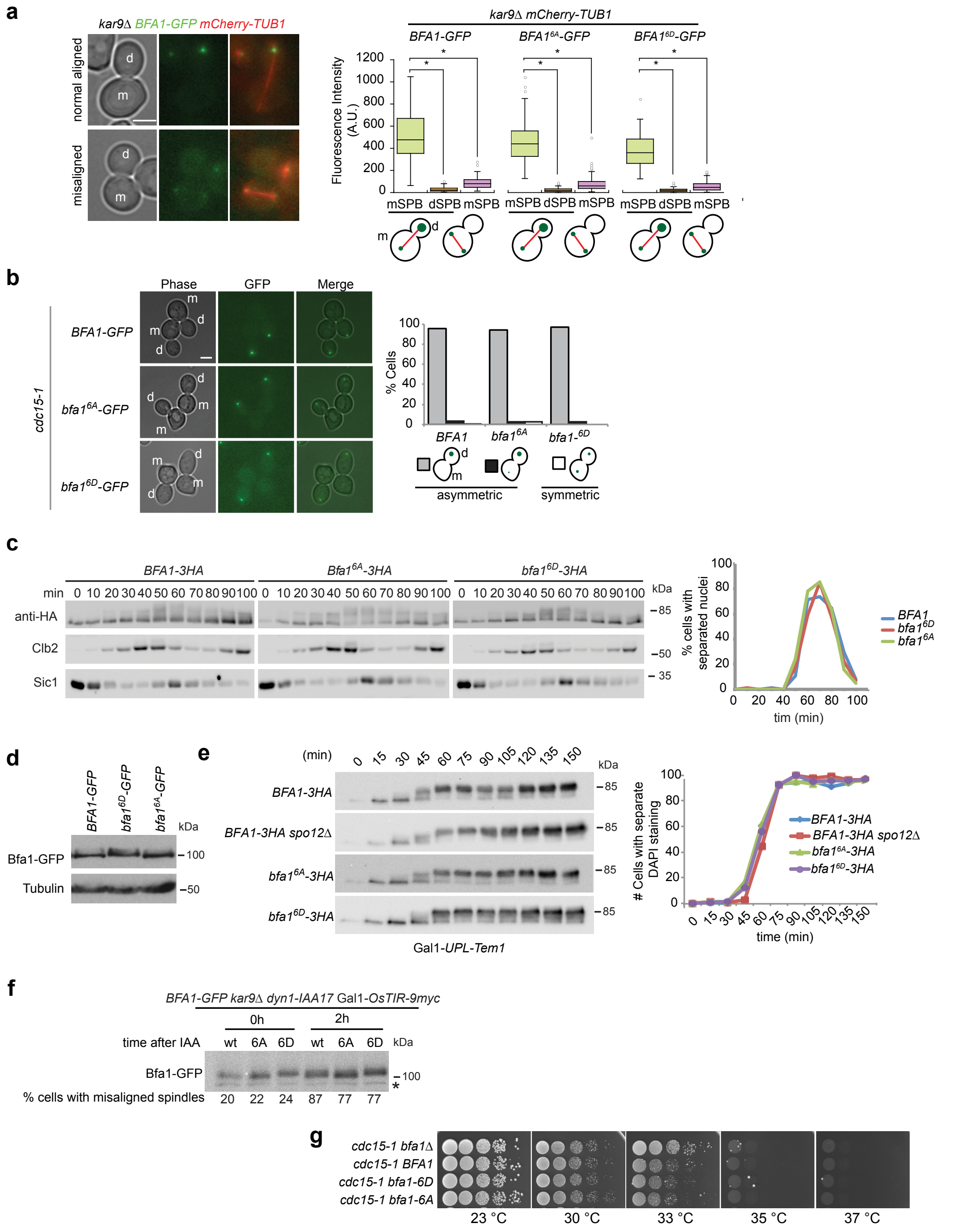

Supplementary Figure 9. Localization, phosphorylation and cell cycle progression of Bfa1 mutants.

a-b. Localization of Bfa1 mutants at the SPBs. (a). SPB localization of Bfa1-GFP, Bfa1-6A-GFP and Bfa1-6D-GFP during spindle normal and misalignment. Tub1-mCherry served as a reference for the spindle position. Representative still images of cells with correctly (on the top) and misaligned (on the bottom) were shown. Mean fluorescence intensities of Bfa1-GFP at the SPBs were shown in the box and whisker plots. The boxes show the lower and upper quartiles, the whiskers show the minimum and maximal values excluding outliers; outliers (shown as dots) were calculated as values greater or lower than 1.5 times the interquartile range; the line inside the box indicates the median. Asterisks mark significant differences

according to two-tailed Student's t-test ($p<0.005$). Numbers of SPBs quantified were 56, 56, 92, 67, 67, 48, 56, 56 and 50 in the order they appear on graph (from left to right). Scale bar: 3 µm. "m" and "d" denote mother and daughter cell compartments respectively.

(b). SPB localization of Bfa1-GFP, Bfa1-6A-GFP and Bfa1-6D-GFP during a late anaphase arrest with correctly positioned spindles. Representative still images are shown on the left. SPB localization was grouped into two categories: asymmetric and symmetric (white bars). Asymmetric localization was also grouped into two: signal absent from the mother SPB (grey, strongly asymmetric), signal present at very low levels at the mother SPB (black, asymmetric). 100 cells were quantified from each strain. Results are from one experiment. Late anaphase arrest was achieved by use of *cdc15-1* temperature sensitive mutant. Cells were arrested in G1 by the mating pheromone alpha factor (to mark the mother cells with the shmoo) and released from the G1 arrest at restrictive temperature (37°C). Scale bar: 3 µm. "m" and "d" denote mother and daughter cell compartments respectively.

c. Cell cycle progression of Bfa1 mutants. Cells were released from G1-block (time point 0) and samples were collected at indicated time points. Immunoblots show Clb2 and Sic1 as cell cycle markers and migration profile of Bfa1-3HA on SDS-PAGE was also analyzed (anti-HA). The graph indicates the percentages of cells with separated nuclei (anaphase) during the time-course. Immunoblot is one representative out of 3 independent experiments.

d. Immunoblots showing expression levels of indicated Bfa1 mutants.

e. Time-course series of the experiment shown in Fig. 6c. Briefly, cultures of indicated strains were arrested in G1 and released under Tem1 depleting conditions. The graph indicates the percentages of cells with separated nuclei (anaphase). 100 cels were counted per strain per time point.

f. Migration profile of Bfa1 mutants in cells with misaligned spindles. *kar9*Δ *DYN1-IAA17* Gal-*OsTIR1-9myc* cells containing GFP tagged wild-type Bfa1 (wt), Bfa1-6A (6A), and Bfa1-6D (6D) were analyzed before addition of IAA (t=0h) and 2 h after addition of IAA (t:2h). Percentage of cells with misaligned spindles are indicated. Note that Bfa1 during spindle misalignment is hypophosphorylated. Bfa1-6D runs slightly slower than Bfa1 or Bfa1-6A.

g. Growth of Bfa1 mutants. Serial dilutions of indicated strains were spotted on YPD plates and incubated at indicated temperatures.

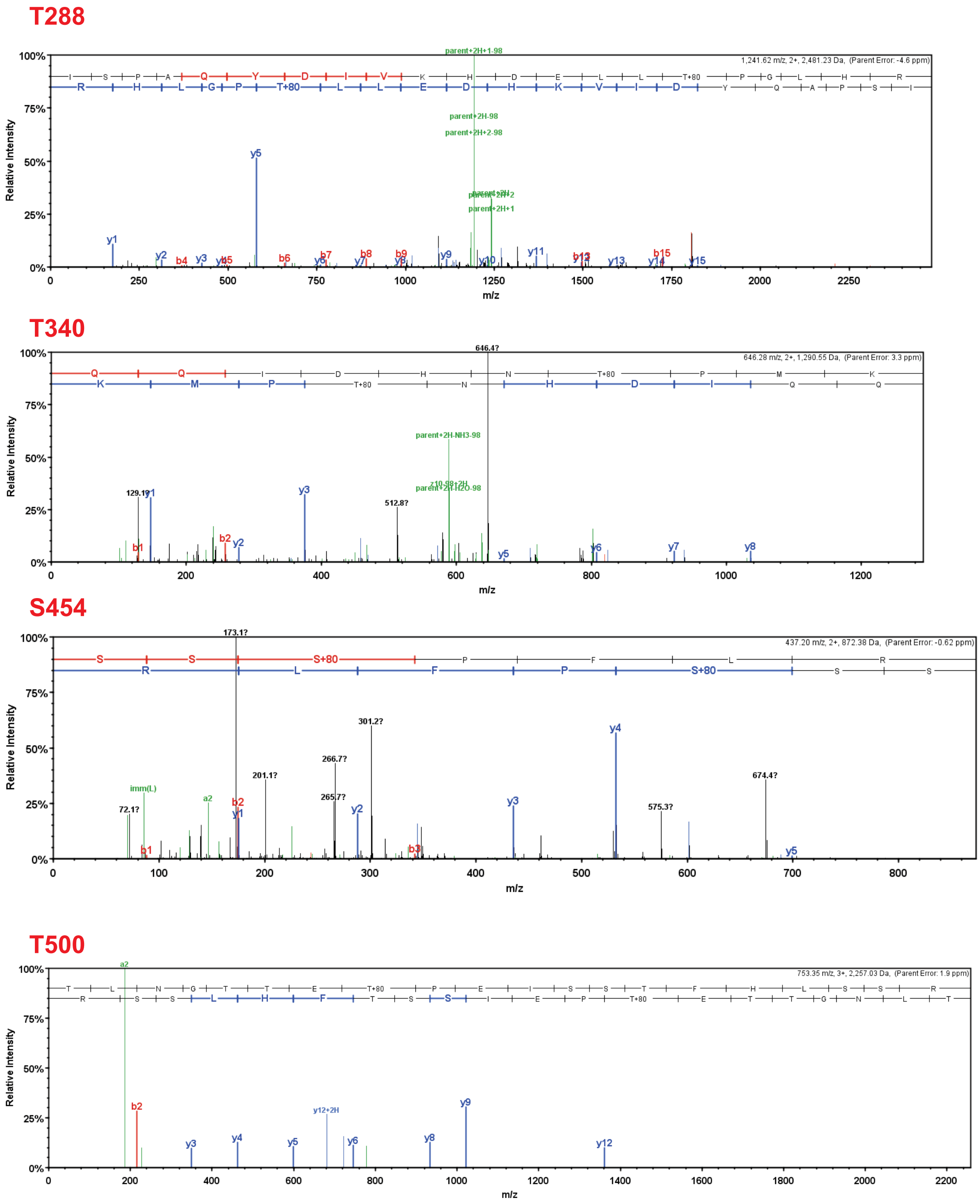

Supplementary Figure 10. Detection of Bfa1 phosphorylation at Cdk consensus sites in vivo. Bfa1 was pulled down from metaphase arrested cells (Cdc20 depletion). MS-MS spectra generated by the mass spectrometer (Scaffold 4 Proteome Software) is shown.

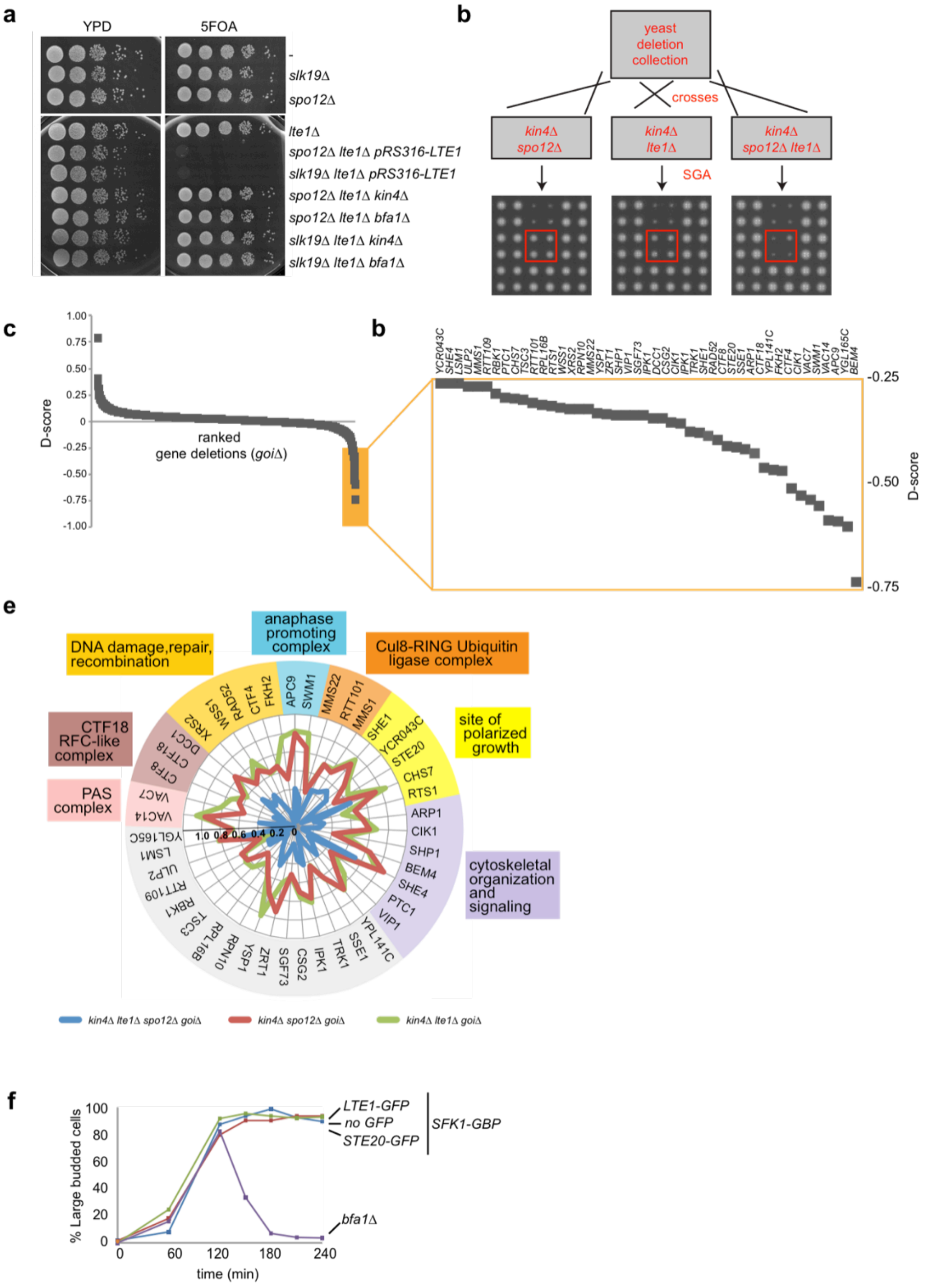

Supplementary Figure 11. Genome-wide screen to identify MEN activators parallel to FEAR and Kin4-independent function of Lte1.

a. Deletion of *KIN4* and *BFA1* rescues *spo12*Δ *lte1*Δ and *slk19*Δ *lte1*Δ lethality.

b. Schematic representation of screen strategy. Yeast deletion library was crossed with the indicated query strains. Examples of the colony growth after haploid selection by the synthetic genetic array (SGA) methodology. Red square marks a gene of interest with the desired growth phenotype.

c-d. D-scores for deletion of each gene of interest (*goi*Δ). Orange box outlines the D-score values lower than -0.25, which are considered as the gene deletions that cause significant growth retardation in *kin4*Δ *spo12*Δ *lte1*Δ cells. D is the zoom-in of the orange box shown in c. D-score: the difference between the colony size of each *kin4*Δ *lte1*Δ *spo12*Δ *goi*Δ cross and the minimum colony size of the two control crosses (*kin4*Δ *lte1*Δ *goi*Δ and *kin4*Δ *spo12*Δ *goi*Δ).

e. Radar graph representing the mean colony sizes (0 to 1.0) of *kin4*Δ *lte1*Δ *spo12*Δ *goi*Δ

(blue circle), *kin4*Δ *spo12*Δ *goi*Δ (red circle) and *kin4*Δ *lte1*Δ *goi*Δ (green circle) cells where *goi*Δ represents the deletion of the gene indicated at the outermost layer. Only the 43 gene deletions considered as hits (see the criteria in Methods section) are shown. The hits are categorized according to the indicated Gene Ontology categories. Different categories are shown in different colors.

f. Ste20 and Lte1 recruitment to the mother cell does not bypass the metaphase arrest exerted by nocodazole treatment. Ste20-GFP and Lte1-GFP were artificially recruited to the mother cell through SFK1-GBP. Graphs represent the percentage of large budded cells counted in a time-course experiment, where indicated cell types were synchronized in G1 (t=0) and released from the G1-block in nocodazole containing medium. *bfa1*Δ serves as a control that is unable to arrest upon microtubule depolymerization.

Fig. 4a

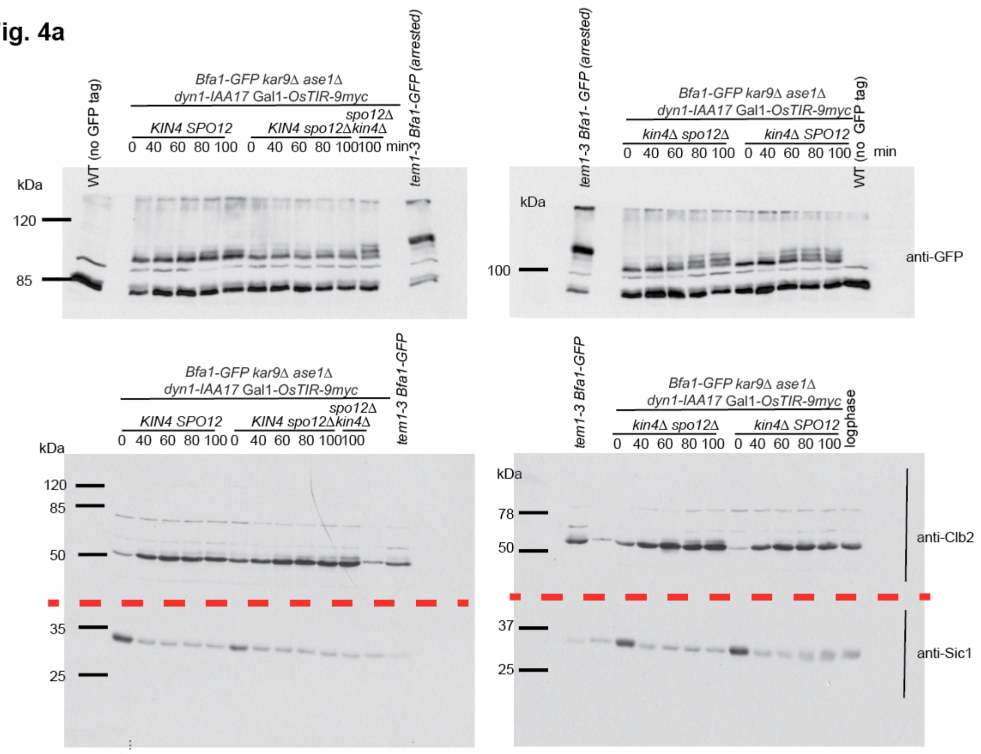

Fig. 4c

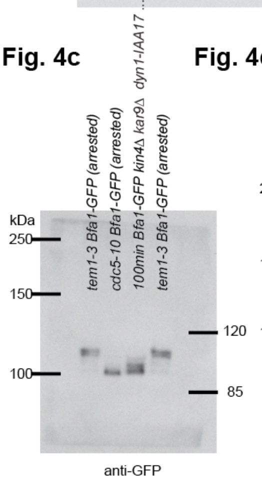

Fig. 4d

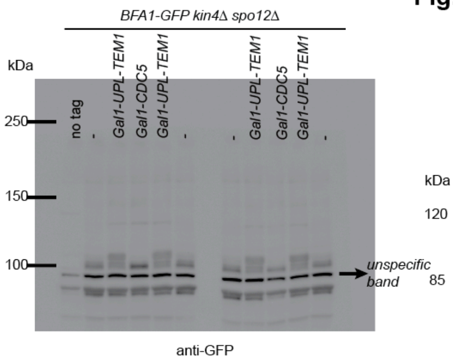

Fig. 4e

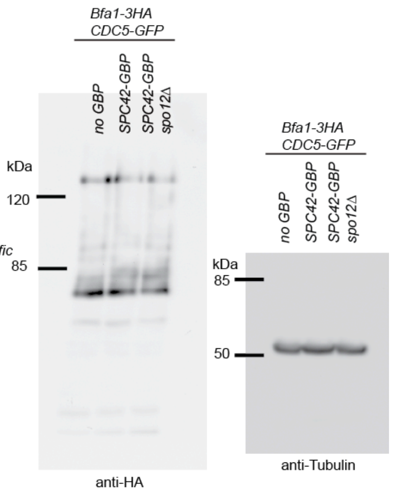

Supplementary Figure 12. Uncut versions of all immunoblots and coomassie stainings of SDS-PAGE gels shown in the entire manuscript. (Continued on next pages)

Fig. 6a

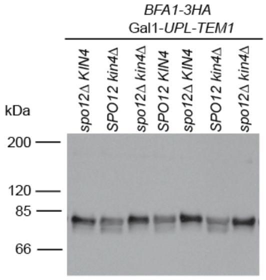

Fig. 6c

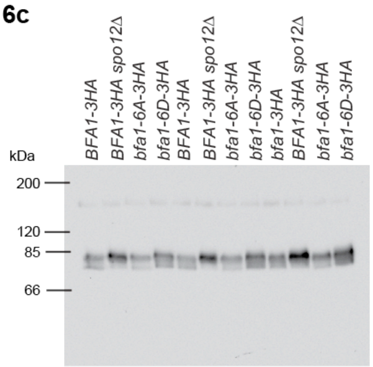

Fig. 6b

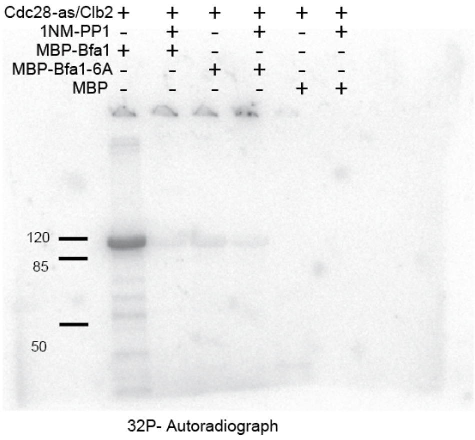

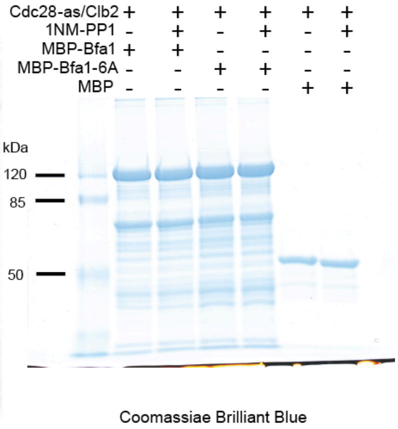

Supplementary Figure 12 (continued)

Supplementary Fig. 5c

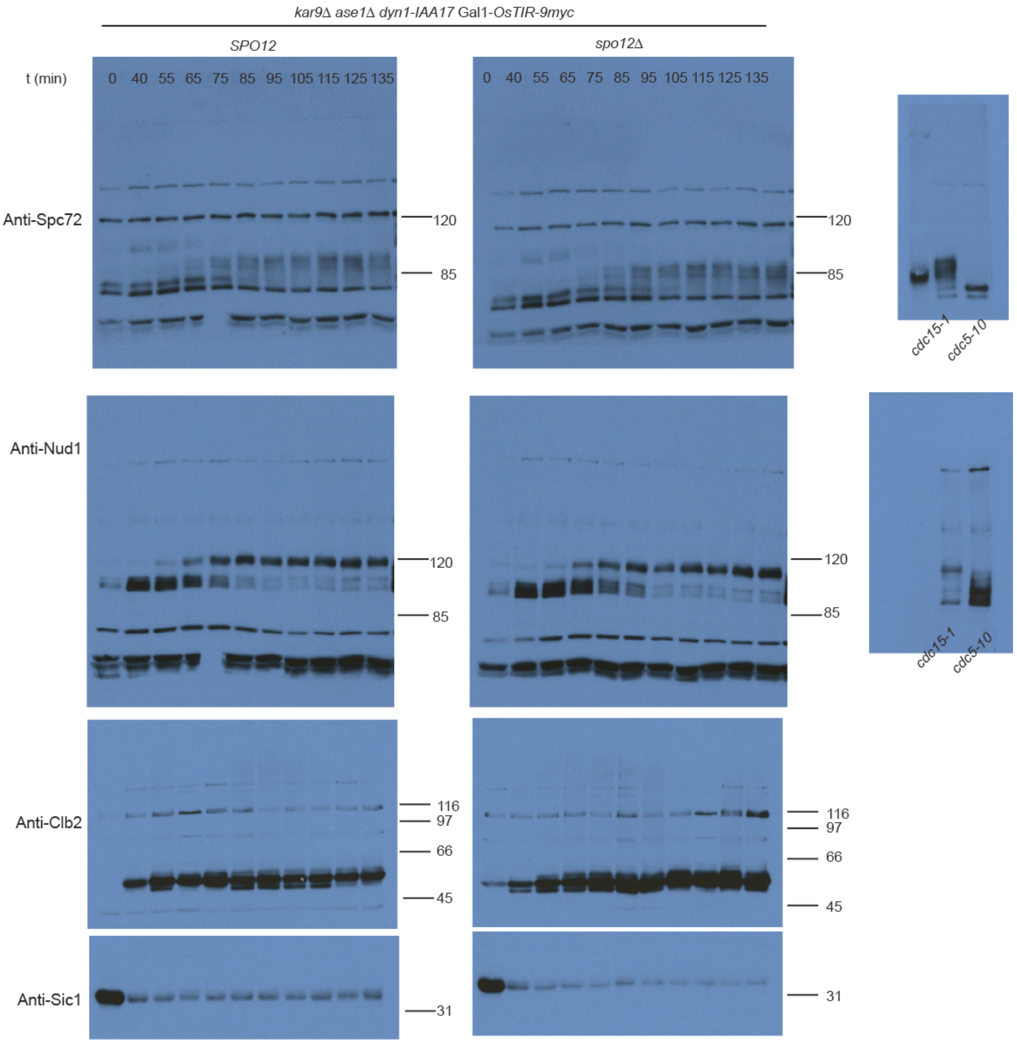

Supplementary Figure 12 (continued)

Supplementary Fig. 6a

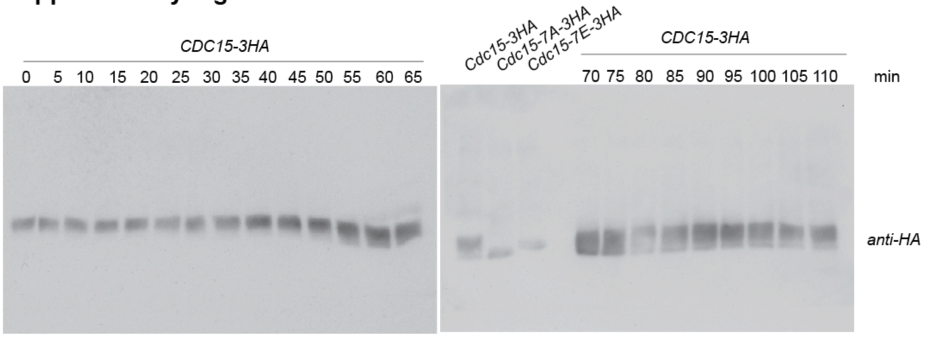

Supplementary Fig. 6b

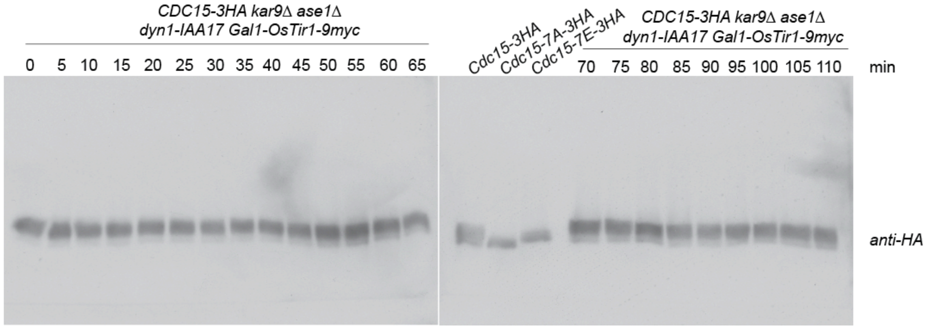

Supplementary Fig. 8

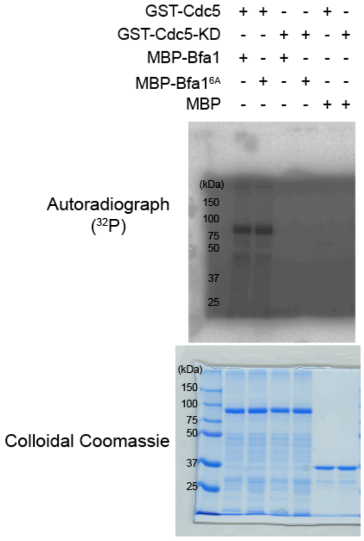

Supplementary Figure 12 (continued)

Supplementary Fig. 9c

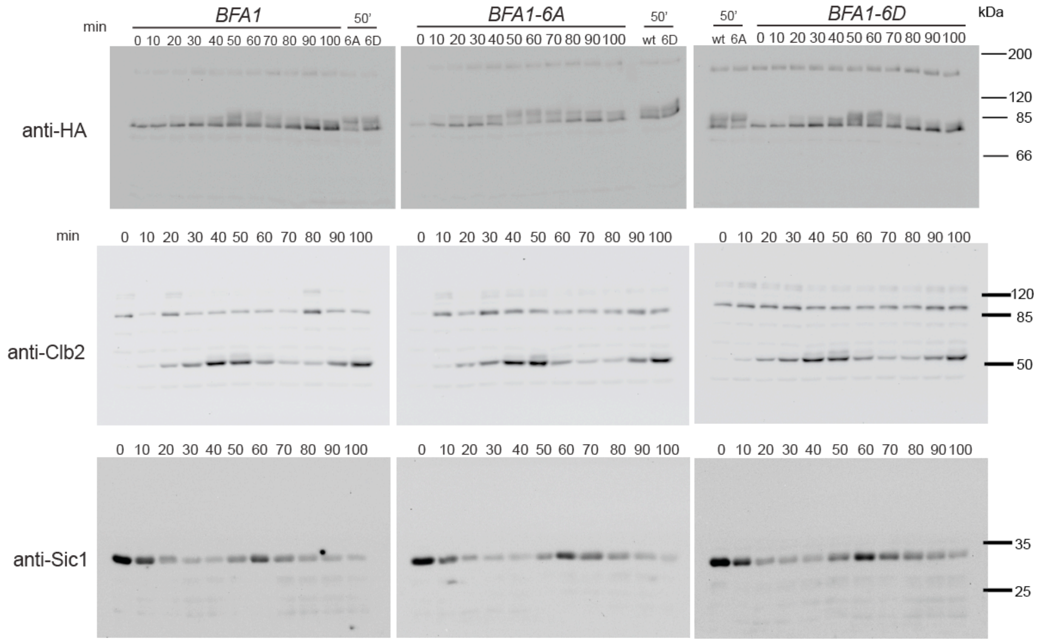

Supplementary Fig. 9d

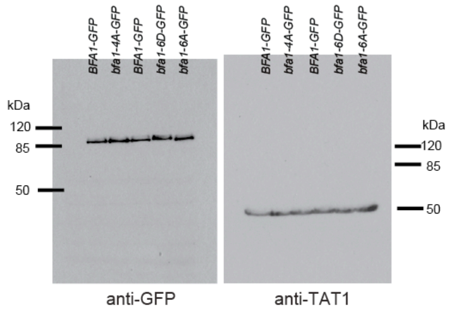

Supplementary Fig. 9e

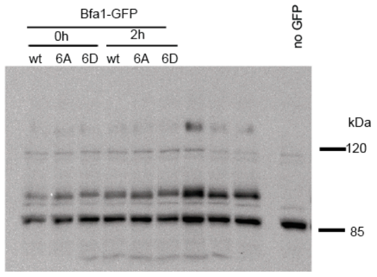

Supplementary Fig. 9f

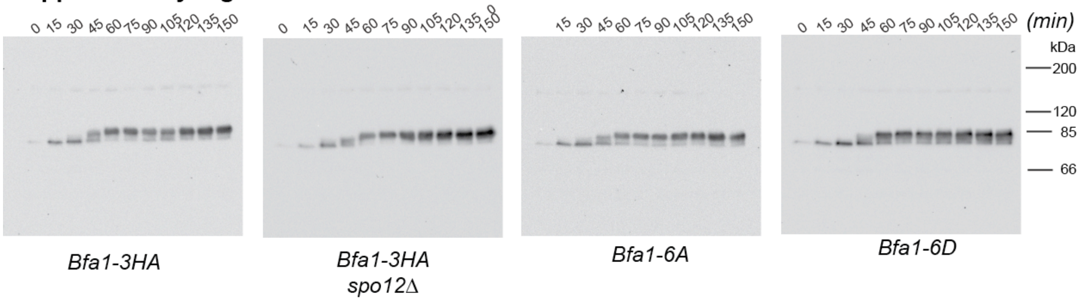

Supplementary Figure 12 (continued)

Supplementary Tables (1-2)

Supplementary Table 1. Table of yeast strains

Yeast strains	***Genotype***	***Source/Reference****
AKY1218	ESM356-1 *SPC42-eqFP-kanMX6 natNT2*-Gal1-*GFP-KIN4 ura3-52::URA3*-Met25-*KIN4 bfa1Δ::klTRP1* pRS315	This Study
AKY1347	ESM356-1 *SPC42-eqFP-kanMX6 natNT2*-Gal1-*GFP-KIN4 ura3-52::URA3*-Met25-*KIN4* pRS315	This Study
AKY1084	ESM356-1 *SPC42-eqFP-kanMX6 natNT2*-Gal1-*GFP-KIN4 ura3-52::URA3*-Met25-*KIN4* pTH20	This Study
AKY1348	ESM356-1 *SPC42-eqFP-kanMX6 natNT2*-Gal1-*GFP-KIN4 ura3-52::URA3*-Met25-*KIN4* pCL004	This Study
AKY2313	*YPH499 kar9Δ::klTRP1 ade2-101::ADE2-GFP-TUB1* pGW399 pRS315	This Study
AKY2314	*YPH499 kar9Δ::klTRP1 ade2-101::ADE2-GFP-TUB1* pGW399 pTH20	This Study
AKY2315	*YPH499 kar9Δ::klTRP1 ade2-101::ADE2-GFP-TUB1* pGW399 pCL004	This Study
AKY260	ESM356-1 *kar9Δ::HIS3MX6* pGW399	This Study
AKY346-1	*YPH499 kar9Δ::klTRP1 ade2-101::ADE2-GFP-TUB1* pGW399	[31]
AKY351-1	*YPH499 kar9Δ::klTRP1 ade2-101::ADE2-GFP-TUB1* pGW399 *kin4Δ::HIS3MX6*	[31]
AKY353-1	*YPH499 kar9Δ::klTRP1 ade2-101::ADE2-GFP-TUB1* pGW399 *rts1Δ::HIS3MX6*	[31]
AKY357-1	*YPH499 kar9Δ::klTRP1 ade2-101::ADE2-GFP-TUB1* pGW399 *bfa1Δ::HIS3MX6*	[31]
YMY390-1	*YPH499 kar9Δ::klTRP1 ade2-101::ADE2-GFP-TUB1* pGW399 *bmh1Δ::HIS3MX6*	[26]
AKY1280-1	*YPH499 kar9Δ::klTRP1 ade2-101::ADE2-GFP-TUB1* pGW399 *spo12Δ::hphNT1*	This Study
AKY1275-1	*YPH499 kar9Δ::klTRP1 ade2-101::ADE2-GFP-TUB1* pGW399 *rts1Δ::HIS3MX6 spo12Δ::hphNT1*	This Study
AKY1277-1	*YPH499 kar9Δ::klTRP1 ade2-101::ADE2-GFP-TUB1* pGW399 *bfa1Δ::HIS3MX6 spo12Δ::hphNT1*	This Study
AKY1279-1	*YPH499 kar9Δ::klTRP1 ade2-101::ADE2-GFP-TUB1* pGW399 *kin4Δ::HIS3MX6 spo12Δ::hphNT1*	This Study
AKY1840-1	*YPH499 kar9Δ::klTRP1 ade2-101::ADE2-GFP-TUB1* pGW399 *bmh1Δ::HIS3MX6 spo12Δ::hphNT1*	This Study
AKY313-1	ESM356-1 *kar9Δ::HIS3MX6 bub2Δ::klTRP1* pGW399	This Study
AKY2619-1	SP4659 *kar9Δ::HIS3MX6*	This Study
AKY2647-1	SP4659 *kar9Δ::hphNT spo12Δ::HIS3MX6*	This Study
AKY2228-1	K699 *kar9Δ::natNT2*	This Study
AKY2230-1	K699 *bub2Δ::hphNT1 kar9Δ::natNT2*	This Study
AKY2229-1	RJD2862 *kar9Δ::natNT2*	This Study
AKY2231-1	RJD2862 *bub2Δ::hphNT1 kar9Δ::natNT2*	This Study
AKY2377-1	K699 *kin4Δ::kanMX6 kar9Δ::klTRP1*	This Study
AKY2288-1	RJD2862 *kin4Δ::kanMX6 kar9Δ::natNT2*	This Study
AKY1321-1	*YPH499 kar9Δ::klTRP1 ade2-101::ADE2-GFP-TUB1* pGW399 *kin4Δ::HIS3MX6 slk19Δ::hphNT1*	This Study
AKY1323-1	*YPH499 kar9Δ::klTRP1 ade2-101::ADE2-GFP-TUB1* pGW399 *slk19Δ::hphNT1*	This Study

AKY2210-1	ESM356-1 *ura3-52*::*URA3*-Gal-*OsTIR1-9myc DYN1-IAA17-kanMX4 kar9Δ::HIS3MX6 leu2Δ1*::*LEU2-mCherry-TUB1 CDC14-GFP-hphNT1*	This Study
AKY2264-1	ESM356-1 *ura3-52*::*URA3*-Gal-*OsTIR1-9myc DYN1-IAA17-kanMX4 kar9Δ::HIS3MX6 leu2Δ1*::*LEU2-mCherry-TUB1 CDC14-GFP-hphNT1 spo12Δ::natNT2*	This Study
AKY2289-1	ESM356-1 *ura3-52*::*URA3*-Gal-*OsTIR1-9myc DYN1-IAA17-kanMX4 kar9Δ::HIS3MX6 leu2Δ1-LEU2-SLI15-GFP-kanMX6 trp1Δ63::klTRP1-mcherry-TUB1*	This Study
AKY2290-1	ESM356-1 *ura3-52*::*URA3*-Gal-*OsTIR1-9myc DYN1-IAA17-kanMX4 kar9Δ::HIS3MX6 leu2Δ1-LEU2-SLI15-GFP-kanMX6 trp1Δ63::klTRP1-mcherry-TUB1 spo12Δ::hphNT1*	This Study
AKY2463-1	ESM356-1 *ura3-52*::*URA3*-Gal-*OsTIR1-9myc DYN1-IAA17-kanMX4 kar9Δ::HIS3MX6 BFA1-GFP-hphNT1 ase1Δ::loxP-klLEU2-loxP*	This Study
AKY2464-1	ESM356-1 *ura3-52*::*URA3*-Gal-*OsTIR1-9myc DYN1-IAA17-kanMX4 kar9Δ::HIS3MX6 BFA1-GFP-hphNT1 ase1Δ::loxP-klLEU2-loxP spo12Δ:natNT2*	This Study
AKY2465-1	ESM356-1 *ura3-52*::*URA3*-Gal-*OsTIR1-9myc DYN1-IAA17-kanMX4 kar9Δ::HIS3MX6 BFA1-GFP-hphNT1 ase1Δ::loxP-klLEU2-loxP spo12Δ:natNT2 kin4Δ::klTRP1*	This Study
AKY2477-1	ESM356-1 *ura3-52*::*URA3*-Gal-*OsTIR1-9myc DYN1-IAA17-kanMX4 kar9Δ::HIS3MX6 BFA1-GFP-hphNT1 ase1Δ::loxP-klLEU2-loxP kin4Δ::klTRP1*	This Study
AKY2655-2	YPH499 *cdc5-10 BFA1-GFP-hphNT1*	This Study
AKY2915- 1	ESM356 *kin4Δ::klTRP1 spo12Δ::natNT2 BFA1-GFP-kanMX6*	This Study
AKY3032-1	ESM356-1 *kin4Δ::klTRP1 spo12Δ::hphNT1 BFA1-GFP-KanMX6 NatNT2*-Gal1-*CDC5*	This Study
AKY2901-1	ESM356-1 *BFA1-GFP-KanMX6 Spc42-eqFP-hphNT1 tem1::*Gal1-UPL1-*TEM1-URA3*	This Study
AKY3073-1	ESM356 *kar9Δ::his3MX6* Cdc5-GFP-hphNT1 *BFA1-3HA-kanMX6* pGW399	This Study
AKY3074-1	ESM356 *SPC42-GBP-kanMX6 kar9Δ::klTRP1 CDC5-GFP-hphNT BFA1-3HA-kanMX6*	This Study
AKY3075-1	ESM356 *SPC42-GBP-kanMX6 kar9Δ::klTRP1 spo12Δ::his3MX6 CDC5-GFP-hphNT spo12Δ::natNT1 BFA1-3HA-kanMX6.*	This Study
AKY3075-1	ESM356 *SPC42-GBP-kanMX6 kar9Δ::klTRP1 spo12Δ::his3MX6 CDC5-GFP-hphNT spo12Δ::natNT1 BFA1-3HA-kanMX6.*	This Study
GPY1142	ESM356 *kar9Δ::his3MX6 CDC5-GFP-HphNT1* pGW399	This Study
AKY2629	ESM356 *SPC42-GBP-kanMX6 kar9Δ::klTRP1 CDC5-GFP-hphNT*	This Study
AKY3047	SM356 *SPC42-GBP-kanMX6 kar9Δ::klTRP1 spo12Δ::his3MX6 CDC5-GFP-hphNT spo12Δ::natNT1*	This Study
AKY2302-1	ESM356-1 *kar9Δ::HIS3MX6 ura3-52::URA3*-Gal-*OsTIR1*-9myc *DYN1-IAA17-kanMX4 MOB1-GFP-hphNT1 leu2Δ1::LEU2-mCherry-TUB1 spo12Δ::natNT2 kin4Δ::klTRP1*	This Study
AKY2304-1	ESM356-1 *kar9Δ::HIS3MX6 ura3-52::URA3*-Gal-*OsTIR1*-9myc *DYN1-IAA17-kanMX4 MOB1-GFP-hphNT1 kin4Δ::klTRP1 leu2Δ1::LEU2-mCherry-TUB1*	This Study
AKY2216-1	ESM356-1 *kar9Δ::HIS3MX6 ura3-52::URA3*-Gal-*OsTIR1*-9myc *DYN1-IAA17-kanMX4 MOB1-GFP-hphNT1 leu2Δ1::LEU2-mCherry-TUB1*	This Study
AKY2267-1	ESM356-1 *kar9Δ::HIS3MX6 ura3-52::URA3*-Gal-*OsTIR1*-9myc *DYN1-IAA17-kanMX4 MOB1-GFP-hphNT1 leu2Δ1::LEU2-mCherry-TUB1 spo12Δ::natNT2*	This Study

AKY2504-1	ESM356-1 *kar9Δ::HIS3MX6 ura3-52::URA3*-Gal-*OsTIR1*-9myc *DYN1-IAA17-kanMX4 leu2Δ1::LEU2-mCherry-TUB1 CDC15-GFP-hphNT1 spo12Δ::natNT2*	This Study
AKY2505-1	ESM356-1 *kar9Δ::HIS3MX6 ura3-52::URA3*-Gal-*OsTIR1*-9myc *DYN1-IAA17-kanMX4 leu2Δ1::LEU2-mCherry-TUB1 CDC15-GFP-hphNT1 spo12Δ::natNT2 kin4Δ::klTRP1*	This Study
AKY2506-1	ESM356-1 *kar9Δ::HIS3MX6 ura3-52::URA3*-Gal-*OsTIR11*-9myc *DYN1-IAA17-kanMX4 leu2Δ1::LEU2-mCherry-TUB1 CDC15-GFP-hphNT1 kin4Δ::klTRP1*	This Study
AKY2507-1	ESM356-1 *kar9Δ::HIS3MX6 ura3-52::URA3*-Gal-*OsTIR11*-9myc *DYN1-IAA17-kanMX4 leu2Δ1::LEU2-mCherry-TUB1 CDC15-GFP-hphNT1*	This Study
AKY2133	ESM356-1 *mob1Δ::klTRP1 leu2Δ1::MOB1-hphNT1 kar9Δ::HIS3MX6* pGW399	This Study
AKY2147-1	ESM356-1 *mob1Δ::klTRP1 leu2Δ1::mob1*^{2A}*-hphNT1 kar9Δ::HIS3MX6* pGW399 *spo12Δ::kanMX6*	This Study
AKY2148-1	ESM356-1 *cdc15*^{7A} *trp1Δ63::klTRP1-mCherry-TUB1 mob1Δ::natNT2 leu2Δ1::MOB1-hphNT1* pGW399 *kar9Δ::HIS3MX6 spo12Δ::kanMX6*	This Study
AKY2149-1	ESM356-1 *cdc15*^{7A} *trp1Δ63::klTRP1-mCherry-TUB1 mob1Δ::natNT2 leu2Δ1::mob1*^{2A}*-hphNT1* pGW399 *kar9Δ::HIS3MX6 spo12Δ::kanMX6*	This Study
AKY2150-1	ESM356-1 *mob1Δ::klTRP1 leu2Δ1::MOB1-hphNT1 kar9Δ::HIS3MX6* pGW399 *spo12Δ::kanMX6*	This Study
AKY2107-1	ESM356-1 *mob1Δ::klTRP1 leu2Δ1::mob1*^{2A}*-hphNT1* pGW399 *kar9Δ::HIS3MX6*	This Study
AKY2108-1	ESM356-1 *cdc15*^{7A} *trp1Δ63::klTRP1-mCherry-TUB1 mob1Δ::natNT2 leu2Δ1::MOB1-hphNT1* pGW399 *kar9Δ::HIS3MX6*	This Study
AKY2109-1	ESM356-1 *cdc15*^{7A} *trp1Δ63::klTRP1-mCherry-TUB1 mob1Δ::natNT2 leu2Δ1::mob1*^{2A}*-hphNT1* pGW399 *kar9Δ::HIS3MX6*	This Study
AKY2822-1	ESM356-1 *cdc15*^{7A} *trp1Δ63::klTRP1-mCherry-TUB1 BFA1-LEU2::bfa1Δ::HIS3MX6 kar9Δ::hphNT1*	This Study
AKY2824-1	ESM356-1 *cdc15*^{7A} *trp1Δ63::klTRP1-mCherry-TUB1 bfa1*^{6A}*-LEU2::bfa1Δ::HIS3MX6 kar9Δ::hphNT1*	This Study
AKY2840-1	ESM356-1 *cdc15*^{7A} *trp1Δ63::klTRP1-mCherry-TUB1 BFA1-LEU2::bfa1Δ::HIS3MX6 kar9Δ::hphNT1 spo12Δ::kanMX6*	This Study
AKY2894-1	ESM356-1 *cdc15*^{7A} *trp1Δ63::klTRP1-mCherry-TUB1 bfa1*^{6A}*-LEU2::bfa1Δ::HIS3MX6 spo12Δ::hphNT1 kar9Δ:: natNT2*	This Study
AKY2490-1	ESM356-1 *BFA1-3HA-hphNT1 tem1::Gal1-UPL1-TEM1-klTRP1*	This Study
AKY2491-1	ESM356-1 *BFA1-3HA-hphNT1 spo12Δ::his3MX6 tem1::Gal1-UPL1-TEM1-klTRP1*	This Study
AKY2492-1	ESM356-1 *BFA1-3HA-hph kin4Δ::his3MX6 spo12Δ::natNT2 tem1::Gal1-UPL1-TEM1-klTRP1*	This Study
AKY2493-1	ESM356-1 *BFA1-3HA-hph kin4Δ::his3MX6 tem1::Gal1-UPL1-TEM1-klTRP1*	This Study
AKY2491-1	ESM356-1 *BFA1-3HA-hphNT1 tem1::*Gal1-UPL1-*TEM1-klTRP1 spo12Δ::HIS3MX6*	This Study
AKY2789-1	ESM356-1 *BFA1-3HA-hphNT1-LEU2::bfa1Δ::klTRP1 tem1::*Gal1-UPL1-*TEM1-URA3*	This Study
AKY2790-1	ESM356-1 *bfa1*^{6D}*-3HA-hphNT1-LEU2::bfa1Δ::klTRP1 tem1::*Gal1-UPL1-*TEM1-URA3*	This Study
AKY2791-1	ESM356-1 *bfa1*^{6A}*-3HA-hphNT1-LEU2::bfa1Δ::klTRP1 tem1::*Gal1-UPL1-*TEM1-URA3*	This Study
AKY314-1	ESM356-1 *bfa1Δ::klTRP1*	This Study
AKY2615-1	ESM356-1 *BFA1-LEU2::bfa1Δ::klTRP1*	This Study
AKY2641-1	ESM356-1 *bfa1*^{6D}*-LEU2::bfa1Δ::klTRP1*	This Study
AKY2642-2	ESM356-1 *bfa1*^{6A}*-LEU2::bfa1Δ::klTRP1*	This Study

AKY315-1	ESM356-1 *kar9Δ::HIS3MX6* pGW399 *bfa1Δ::klTRP1*	This Study
AKY2649-1	ESM356-1 *BFA1-LEU2::bfa1Δ::klTRP1 kar9Δ::hphNT1*	This Study
AKY2664-1	ESM356-1 *bfa1*^{6D}*-LEU2::bfa1Δ::klTRP1 kar9Δ::hphNT1*	This Study
AKY2665-1	ESM356-1 *bfa1*^{6A}*-LEU2::bfa1Δ::klTRP1 kar9Δ::hphNT1*	This Study
AKY2696-1	ESM356-1 *BFA1-LEU2::bfa1Δ::klTRP1 kar9Δ::hphNT1 kin4Δ::HIS3MX6*	This Study
AKY2700-1	ESM356-1 *bfa1*^{6A}*-LEU2::bfa1Δ::klTRP1 kar9Δ::hphNT1 kin4Δ::HIS3MX6*	This Study
AKY2699-1	ESM356-1 *bfa1*^{6D}*-LEU2::bfa1Δ::klTRP1 kar9Δ::hphNT1 kin4Δ::HIS3MX6*	This Study
AKY2701-1	ESM356-1 *BFA1-LEU2::bfa1Δ::klTRP1 kar9Δ::hphNT1 spo12Δ::HIS3MX6*	This Study
AKY2704-1	ESM356-1 *bfa1*^{6D}*-LEU2::bfa1Δ::klTRP1 kar9Δ::hphNT1 spo12Δ::HIS3MX6*	This Study
AKY2705-1	ESM356-1 *bfa1*^{6A}*-LEU2::bfa1Δ::klTRP1 kar9Δ::hphNT1 spo12Δ::HIS3MX6*	This Study
AKY2742-1	ESM356-1 *BFA1-LEU2::bfa1Δ::klTRP1 spo12Δ::HIS3MX6 kin4Δ::hphNT1 kar9Δ::natNT2*	This Study
AKY2745-1	ESM356-1 *bfa1*^{6D}*-LEU2::bfa1Δ::klTRP1 spo12Δ::HIS3MX6 kin4Δ::hphNT1 kar9Δ::natNT2*	This Study
AKY2746-1	ESM356-1 *bfa1*^{6A}*-LEU2::bfa1Δ::klTRP1 spo12Δ::HIS3MX6 kin4Δ::hphNT1 kar9Δ::natNT2*	This Study
AKY696-1	YPH499 *cdc5-10* pRS316-*CDC5 bfa1Δ::HIS3MX6.*	This Study
AKY3072-1	YPH499 *cdc5-10* pRS316-*CDC5 bfa1-6cdk-A-LEU2::bfa1Δ::his3MX6*	This Study
AKY3071-1	YPH499 *cdc5-10* pRS316-*CDC5 bfa1-6cdk-D-LEU2::bfa1Δ::his3MX6*	This Study
AKY3068-1	YPH499 *cdc5-10* pRS316-*CDC5 BFA1-LEU2::bfa1Δ::his3MX6*	This Study
AKY3058	ESM356 *rts1Δ::klTRP1 BFA1-LEU2::bfa1Δ::his3MX6* p426	This Study
AKY3059	ESM356 *rts1Δ::klTRP1 BFA1-LEU2::bfa1Δ::his3MX6* pGW412	This Study
AKY3055	ESM356 *rts1Δ::klTRP1 bfa1-6cdk-D-LEU2::bfa1Δ::his3MX6* p426	This Study
AKY3061	ESM356 *rts1Δ::klTRP1 bfa1-6cdk-A-LEU2::bfa1Δ::his3MX6* p426	This Study
AKY3062	ESM356 *rts1Δ::klTRP1 bfa1-6cdk-A-LEU2::bfa1Δ::his3MX6 PGW412*	This Study
AKY3056	ESM356 *rts1Δ::klTRP1 bfa1-6cdk-D-LEU2::bfa1Δ::his3MX6* pGW412	This Study
RDY61	ESM356 BFA1-GFP-hphNT1-LEU2::bfa1Δ::klTRP1 kar9Δ::his3MX6 pGW399	This Study
AKY3054-1	ESM356 *bfa1-6cdk-A-GFP-hphNT1-LEU2::bfa1Δ::klTRP1 mCherry-Tub1-URA3::ura3 kar9Δ::his3MX6*	This Study
RDY63	ESM356 *BFA1-4cdk-A-GFP-hphNT1-LEU2::bfa1Δ::klTRP1 kar9Δ::his3MX6 pGW399*	This Study
DLY381-1	YPH499 *LTE1-GFP-HIS3MX6 SFK1-GBP-KanMX6 kar9Δ::klTRP1* pGW399	[20]
DLY379-1	YPH499 *LTE1-GFP-HIS3MX6 kar9Δ::klTRP1* pGW399	[20]
DLY425-1	YPH499 *SFK1-GBP-KanMX6 kar9Δ::klTRP1* pGW399	[20]
AKY1960-1	YPH499 *SFK1-GBP-KanMX6 kar9Δ::klTRP1* pGW399 *kin4Δ::hphNT1*	This Study
AKY1961-1	YPH499 *LTE1-GFP-HIS3MX6 SFK1-GBP-KanMX6 kar9Δ::klTRP1* pGW399 *spo12Δ::natNT2 kin4Δ::hphNT1*	This Study
AKY1963-1	YPH499 *SFK1-GBP-KanMX6 kar9Δ::klTRP1* pGW399 *spo12Δ::natNT2*	This Study
AKY1925-1	YPH499 *LTE1-GFP-HIS3MX6 SFK1-GBP-KanMX6 kar9Δ::klTRP1* pGW399 *kin4Δ::hphNT1*	This Study
AKY1926-1	YPH499 *LTE1-GFP-HIS3MX6 SFK1-GBP-KanMX6 kar9Δ::klTRP1* pGW399 *spo12Δ::natNT2*	This Study

AKY2041-1	YPH499 *LTE1-GFP-HIS3MX6 kar9Δ::klTRP1* pGW399 *spo12Δ::kanMX6*	This Study
AKY2042-1	YPH499 *SFK1-GBP-KanMX6 kar9Δ::klTRP1* pGW399 *spo12Δ::natNT2 kin4Δ::hphNT1*	This Study
AKY2043-1	YPH499 *LTE1-GFP-HIS3MX6 kar9Δ::klTRP1* pGW399 *kin4Δ::hphNT*	This Study
AKY2057-1	YPH499 *LTE1-GFP-HIS3MX6 kar9Δ::klTRP1* pGW399 *spo12Δ::kanMX6 kin4Δ::hphNT1*	This Study
AKY2830-1	YPH499 *kar9Δ::klTRP1* pGW399 *SFK1-GBP-KanMX6 lte1Δ::HIS3MX6 leu2Δ1::LEU2::lte1*^{K1273E}*-GFP*	This Study
AKY2831-1	YPH499 *kar9Δ::klTRP1* pGW399 *SFK1-GBP-KanMX6 lte1Δ::HIS3MX6 leu2Δ1::LEU2::LTE1-GFP*	This Study
AKY2832-1	YPH499 *kar9Δ::klTRP1* pGW399 *SFK1-GBP-KanMX6 lte1Δ::HIS3MX6 leu2Δ1::LEU2::lte1*^{F1387E}*-GFP*	This Study
AKY2884-1	YPH499 *kar9Δ::klTRP1* pGW399 *SFK1-GBP-KanMX6 lte1Δ::HIS3MX6 leu2Δ1::LEU2::LTE1-GFP spo12Δ::natNT2 kin4Δ::hphNT1*	This Study
AKY2885-1	YPH499 *kar9Δ::klTRP1* pGW399 *SFK1-GBP-KanMX6 lte1Δ::HIS3MX6 leu2Δ1::LEU2::lte1*^{K1273E}*-GFP spo12Δ::natNT2 kin4Δ::hphNT1*	This Study
AKY2886-1	YPH499 *kar9Δ::klTRP1* pGW399 *SFK1-GBP-KanMX6 lte1Δ::HIS3MX6 leu2Δ1::LEU2::lte1*^{F1387E}*-GFP spo12Δ::natNT2 kin4Δ::hphNT1*	This Study
AKY3048-1	YPH499 *sst1Δ ras1Δ::klTRP ras2Δ::His3MX6 pRS426-TPK1 kar9Δ::natNT.*	This Study
AKY3049-1	YPH499 *sst1Δ ras1Δ::klTRP ras2Δ::His3MX6 pRS426-TPK1 SFK1-GBP-KanMX6 LTE1-GFP-hphNT1 kar9Δ::natNT1*	This Study
AKY3050-1	YPH499 *sst1Δ ras1Δ::klTRP ras2Δ::His3MX6 pRS426-TPK1 LTE1-GFP-hphNT kar9Δ::natNT1*	This Study
AKY3051-1	YPH499 *sst1Δ ras1Δ::klTRP1 ras2Δ::His3MX6 pRS426-TPK1 SFK1-GBP-KanMX6 kar9Δ::natNT1*	This Study
AKY2549-1	YPH499 *SFK1-GBP-KanMX6 kar9Δ::klTRP1* pGW399 *spo12Δ::natNT2 kin4Δ::hphNT1 BEM4-GFP-HIS3MX6*	This Study
AKY2550-1	YPH499 *SFK1-GBP-KanMX6 kar9Δ::klTRP1* pGW399 *spo12Δ::natNT2 kin4Δ::hphNT1 RTS1-GFP-HIS3MX6*	This Study
AKY2551	YPH499 *SFK1-GBP-KanMX6 kar9Δ::klTRP1* pGW399 *spo12Δ::natNT2 kin4Δ::hphNT1 STE20-GFP-HIS3MX6*	This Study
AKY2578-1	YPH499 *SFK1-GBP-KanMX6 kar9Δ::klTRP1* pGW399 *spo12Δ::natNT2 kin4Δ::hphNT1 CIK1-GFP-HIS3MX6*	This Study
AKY2579-1	YPH499 *SFK1-GBP-KanMX6 kar9Δ::klTRP1* pGW399 *spo12Δ::natNT2 kin4Δ::hphNT1 VIP1-GFP-HIS3MX6*	This Study
AKY2580-1	YPH499 *SFK1-GBP-KanMX6 kar9Δ::klTRP1* pGW399 *spo12Δ::natNT2 kin4Δ::hphNT1 SHE4-GFP-HIS3MX6*	This Study
AKY2581-1	YPH499 *SFK1-GBP-KanMX6 kar9Δ::klTRP1* pGW399 *spo12Δ::natNT2 kin4Δ::hphNT1 SHP1-GFP-HIS3MX6*	This Study
AKY2583-1	YPH499 *SFK1-GBP-KanMX6 kar9Δ::klTRP1* pGW399 *spo12Δ::natNT2 kin4Δ::hphNT1 PTC1-GFP-HIS3MX6*	This Study
AKY2584-1	YPH499 *SFK1-GBP-KanMX6 kar9Δ::klTRP1* pGW399 *spo12Δ::natNT2 kin4Δ::hphNT1 CHS7-GFP-HIS3MX6*	This Study
AKY2582-1	YPH499 *SFK1-GBP-KanMX6 kar9Δ::klTRP1 pGW399 spo12Δ::natNT2 kin4Δ::hphNT1 SHE1-GFP-HIS3MX6*	This Study
AKY2552	YPH499 *SFK1-GBP-KanMX6 kar9Δ::klTRP1 pGW399 spo12Δ::natNT2 kin4Δ::hphNT1 YPL141C1-GFP-HIS3MX6*	This Study
AKY2557-1	YPH499 SFK1-GBP-KanMX6 kar9Δ::klTRP1 pGW399 PTC1-GFP-HIS3MX6	This Study
AKY2559-1	YPH499 *SFK1-GBP-KanMX6 kar9Δ::klTRP1* pGW399 *SHE4-GFP-HIS3MX6*	This Study
AKY2560-1	YPH499 *SFK1-GBP-KanMX6 kar9Δ::klTRP1* pGW399 *CIK1-GFP-HIS3MX6*	This Study
AKY2562-1	YPH499 *SFK1-GBP-KanMX6 kar9Δ::klTRP1* pGW399 *VIP1-GFP-HIS3MX6*	This Study
AKY2563-1	YPH499 *SFK1-GBP-KanMX6 kar9Δ::klTRP1* pGW399 *SHP1-GFP-HIS3MX6*	This Study
AKY2534-1	YPH499 *SFK1-GBP-KanMX6 kar9Δ::klTRP1* pGW399 *BEM4-GFP-HIS3MX6*	This Study

AKY2535-1	YPH499 *SFK1-GBP-KanMX6 kar9Δ::klTRP1* pGW399 *RTS1-GFP-HIS3MX6*	This Study
AKY2536-1	YPH499 *SFK1-GBP-KanMX6 kar9Δ::klTRP1* pGW399 *STE20-GFP-HIS3MX6*	This Study
AKY2902	ESM356-1 *ura3::URA3-mcherry-TUB1 STE20-GFP-HIS3MX6*	This Study
YDA159	ESM356-9 *spo12Δ::natNT1* pAKC101 *ste20Δ::hphNT1*	This Study
AKY2526-1	ESM356-9 *lte1Δ::kanMX6* pSM903-4 *spo12Δ::natNT2*	This Study
AKY2594	ESM356-9 *lte1Δ::kanMX6 spo12Δ::natNT2 kin4Δ::HIS3MX6* pSM903-4	This Study
AKY2816	ESM356-9 *lte1Δ::kanMX6 spo12Δ::natNT2 kin4Δ::HIS3MX6* pSM903-4 *ste20Δ::hphNT1* pRS315	This Study
AKY2817	ESM356-9 *lte1Δ::kanMX6 spo12Δ::natNT2 kin4Δ::HIS3MX6* pSM903-4 *ste20Δ::hphNT1* pMF642-2	This Study
AKY2818	ESM356-9 *lte1Δ::kanMX6 spo12Δ::natNT2 kin4Δ::HIS3MX6* pSM903-4 *ste20Δ::hphNT1* pTH35	This Study
AKY2820-1	ESM356-9 *lte1Δ::kanMX6 spo12Δ::natNT2 kin4Δ::HIS3MX6* pSM903-4 *ste11Δ::hphNT1*	This Study
AKY2819-1	ESM356-9 *lte1Δ::kanMX6 spo12Δ::natNT2 kin4Δ::HIS3MX6* pSM903-4 *hog1Δ::hphNT1*	This Study
AKY2813-1	ESM356-9 *lte1Δ::kanMX6 spo12Δ::natNT2 kin4Δ::HIS3MX6* pSM903-4 *kss1Δ::hphNT1*	This Study
AKY2829-1	ESM356-9 *lte1Δ::kanMX6 spo12Δ::natNT2 kin4Δ::HIS3MX6* pSM903-4 *fus3Δ::hphNT1*	This Study
YDA138-1	ESM356-9 *lte1Δ::kanMX6 spo12Δ::natNT2 kin4Δ::HIS3MX6* pSM903-4 *ste20Δ::hphNT1*	This Study
AKY2546	YPH499 *SFK1-GBP-KanMX6 kar9Δ::klTRP1* pGW399 *spo12Δ::natNT2 STE20-GFP-HIS3MX6*	This Study
AKY2833-1	YPH499 *SFK1-GBP-KanMX6 kar9Δ::klTRP1* pGW399 *STE20-GFP-HIS3MX6 fus3Δ::hphNT1*	This Study
AKY2834-1	YPH499 *SFK1-GBP-KanMX6 kar9Δ::klTRP1* pGW399 *STE20-GFP-HIS3MX6 ste11Δ::hphNT1*	This Study
AKY2837-1	YPH499 *SFK1-GBP-KanMX6 kar9Δ::klTRP1* pGW399 *STE20-GFP-HIS3MX6 kss1Δ::hphNT1*	This Study
AKY2838-2	YPH499 *SFK1-GBP-KanMX6 kar9Δ::klTRP1* pGW399 *STE20-GFP-HIS3MX6 hog1Δ::hphNT1*	This Study
ESM1192-19	ESM356-9 *lte1Δ::kanMX6*	[20]
YDA139-1	ESM356-9 *lte1Δ::kanMX6 spo12Δ::natNT2 bfa1Δ::HIS3MX6* pSM903-4 *ste20Δ::hphNT1*	This Study
AKY2914	ESM356-9 *lte1Δ::kanMX6* pSM903-4 *spo12Δ::natNT2 ste20Δ::hphNT1*	This Study
AKY1955	ESM356 *ura3-52::URA3*-Gal1-*BFA1* pAKC115	This Study
AKY1953	ESM356 *ura3-52::URA3*-Gal1-*BFA1* pRS315	This Study
AKY1622	ESM356 *ura3-52::URA3*-Gal1-*BFA1* pRS425	This Study
AKY1636	ESM356 *ura3-52::URA3*-Gal1-*BFA1* pTH20	This Study
ESM1309	YPH499 *dbf2-2*	Gift from Elmar Schiebel
ESM1249	YPH499 *tem1-3*	Gift from Elmar Schiebel
ESM1278	YPH499 *cdc15-1*	[56]
ESM1276	YPH499 cdc14-2	[57]
ESM1361	YPH499 mob1-67	Gift from Elmar Schiebel

CLY169	YPH499 *cdc5-10*	[22]
AKY2470-1	YPH499 *dbf2Δ::kanMX6 dbf20Δ::klTRP1* pSM912	This Study
GPY394	YPH499 *dbf2Δ::kanMX6* pSM912	This Study
SHM1799-1	ESM356-1 *cdc15Δ::klTRP1* pBS9	Gift from Elmar Schiebel
CKY329-3	ESM356-1 *mob1Δ::klTRP1* pSM926	Gift from Elmar Schiebel
GPY120	YPH501 *tem1Δ::kanMX6* pRS316-*TEM1*	This Study
GPY397-1	ESM356-1 *cdc14Δ::kanMX6* pUG120	This Study
CLY130	YPH499 *cdc5Δ::kanMX6* pCL33	Gift from Elmar Schiebel
AKY2217-1	ESM356-1 *ura3-52*::*URA3*-Gal-*OsTIR11-9myc DYN1-IAA17-kanMX4 kar9Δ::HIS3MX6 leu2Δ1*::*LEU2-mCherry-TUB1 NET1-GFP-hphNT1*	This Study
AKY2269-1	ESM356-1 *ura3-52*::*URA3*-Gal-*OsTIR1-9myc DYN1-IAA17-kanMX4 kar9Δ::HIS3MX6 leu2Δ1*::*LEU2-mCherry-TUB1 spo12Δ::natNT2 NET1-GFP-hphNT1*	This Study
AKY2297-1	ESM356-1 *ura3-52*::*URA3*-Gal-*OsTIR1-9myc DYN1-IAA17-kanMX4 kar9Δ::HIS3MX6 leu2Δ1*::*LEU2-mCherry-TUB1 spo12Δ::natNT2 kin4Δ::klTRP1 NET1-GFP-hphNT1*	This Study
AKY2692-1	YPH499 *kar9Δ::klTRP1* pGW399 *ade2-101::ADE2-GFP-TUB1 ase1Δ::hphNT1*	This Study
AKY2693-1	YPH499 *kar9Δ::klTRP1* pGW399 *ade2-101::ADE2-GFP-TUB1 kin4Δ::HIS3MX6 ase1Δ::hphNT1*	This Study
AKY2694-1	YPH499 *kar9Δ::klTRP1* pGW399 *ade2-101::ADE2-GFP-TUB1 kin4Δ::HIS3MX6* cin8Δ::hphNT1.	This Study
AKY2695-1	YPH499 *kar9Δ::klTRP1* pGW399 *ade2-101::ADE2-GFP-TUB1 cin8Δ::hphNT1*	This Study
AKY2476-1	ESM356-1 *ura3-52*::*URA3*-Gal-*OsTIR1-9myc DYN1-IAA17-kanMX4 kar9Δ::HIS3MX6 leu2Δ1*::*LEU2-mCherry-TUB1 CDC5-GFP-hphNT1*	This Study
AKY2482-1	ESM356-1 *ura3-52*::*URA3*-Gal-*OsTIR1-9myc DYN1-IAA17-kanMX4 kar9Δ::HIS3MX6 leu2Δ1*::*LEU2-mCherry-TUB1 spo12Δ::natNT2 CDC5-GFP-hphNT1*	This Study
AKY2352-1	ESM356-1 *ura3-52*::*URA3*-Gal-*OsTIR1-9myc DYN1-IAA17-kanMX4 kar9Δ::HIS3MX6 ase1Δ::klTRP1 CDC15-3HA-hphNT1*	This Study
AKY2390-1	ESM356-1 *ura3-52*::*URA3*-Gal-*OsTIR1-9myc DYN1-IAA17-kanMX4 kar9Δ::HIS3MX6 ase1Δ::klTRP1 CDC15-3HA-hphNT1 spo12Δ::natNT2*	This Study
AKY2212-1	ESM356-1 *ura3-52*::*URA3*-Gal-*OsTIR1-9myc DYN1-IAA17-kanMX4 kar9Δ::HIS3MX6 leu2Δ1*::*LEU2-mCherry-TUB1 BFA1-GFP-hphNT1*	This Study
AKY2263-1	ESM356-1 *ura3-52*::*URA3*-Gal-*OsTIR1-9myc DYN1-IAA17-kanMX4 kar9Δ::HIS3MX6 leu2Δ1*::*LEU2-mCherry-TUB1 BFA1-GFP-hphNT1 spo12Δ::natNT2*	This Study
AKY2300-1	ESM356-1 *ura3-52*::*URA3*-Gal-*OsTIR1-9myc DYN1-IAA17-kanMX4 kar9Δ::HIS3MX6 leu2Δ1*::*LEU2-mCherry-TUB1 BFA1-GFP-hphNT1 spo12Δ::natNT2 kin4Δ::klTRP1*	This Study
AKY2306-1	ESM356-1 *ura3-52*::*URA3*-Gal-*OsTIR1-9myc DYN1-IAA17-kanMX4 kar9Δ::HIS3MX6 leu2Δ1*::*LEU2-mCherry-TUB1 BFA1-*	This Study

	GFP-hphNT1 kin4Δ::klTRP1	
SHM1887-1	ESM356-1 *cdc15Δ::HIS3MX6 CDC15-3HA-LEU2 trp1::TRP1-mCherry-TUB1*	[58]
SHM1888-16	ESM356-1 cdc15Δ::His3MX6 CDC15-7A-3HA-LEU2 trp1::TRP1-mCherry-TUB1	[58]
SHM1889-10	ESM356-1 cdc15Δ::His3MX6 CDC15-7E-3HA-LEU2 trp1::TRP1-mCherry-TUB1	[58]
AKY2352-1	ESM356 *kar9Δ::his3MX6 ura3::URA3-Gal1-OsTIR-9myc DYN1-IAA17-kanMX4 ase1Δ::klTRP CDC15-3HA-hphNT1.*	This Study
AKY935-1	ESM356 *CDC15-3HA-His3MX6.*	This Study
AKY3038-1	YPH499 *cdc15-1 Δbfa1::klTRP1 bfa1-6cdk-A-GFP-HIS3MX6-LEU2::bfa1Δ::his3MX6*	This Study
AKY3039-1	YPH499 *cdc15-1 BFA1-GFP-HIS3MX6-LEU2::bfa1Δ::klTRP1*	This Study
AKY3041-1	YPH499 *cdc15-1 bfa1-6cdk-D-GFP-HIS3MX6-LEU2::bfa1Δ::klTRP1*	This Study
AKY3052-1	ESM356 *BFA1-GFP-hphNT-LEU2::bfa1Δ::klTRP1 ura3::URA3-mCherry-TUB1 kar9Δ::his3MX6.*	This Study
AKY3053-1	ESM356 *bfa1-6cdk-D-LEU2::bfa1Δ::klTRP1 Bfa1-GFP-hphNT1 ura3::URA3-mCherry-TUB1 kar9kar9Δ::his3MX6*	This Study
AKY3054-1	ESM356 *bfa1-6cdk-A-LEU2::bfa1Δ::klTRP1 Bfa1-GFP-hphNT1 ura3::URA3-mCherry-TUB1 kar9kar9Δ::his3MX6*	This Study
AKY2169	ESM356 *kar9Δ::his3MX6 ura3::URA3-Gal1-OsTIR9myc DYN1-IAA17-kanMx4 BFA1-GFP-hphNT1*	This Study
RDY50-1	EMS356 *kar9Δ::natNT2 ura3::URA3-Gal1-OsTIR9myc DYN1-IAA17-kanMx4 bfa1-6cdk-A-GFP-hphNT1-LEU2::bfa1Δ::KlTRP1*	This Study
RDY49-1	EMS356 *kar9Δ::natNT2 ura3::URA3-Gal1-OsTIR9myc DYN1-IAA17-kanMx4 bfa1-6cdk-D-GFP-hphNT1-LEU2::bfa1Δ::KlTRP1*	This Study
AKY2621-1	ESM356-1 *BFA1-GFP-hphNT1-LEU2::bfa1Δ::klTRP1*	This Study
AKY2671-1	ESM356-1 *$bfa1^{6D}$-GFP-hphNT1-LEU2::bfa1Δ::klTRP1*	This Study
AKY2672-1	ESM356-1 *$bfa1^{6A}$-GFP-hphNT1-LEU2::bfa1Δ::klTRP1*	This Study
AKY2399-1	Y8205 pTH17 slk19Δ::natNT2	This Study
AKY2400-2	Y8205 pTH17 *spo12Δ::natNT2*	This Study
AKY2367-1	Y8205 *lte1Δ::hphNT1*	This Study
AKY2397-1	Y8205 *lte1Δ::hphNT1* pTH17 *spo12Δ::natNT2*	This Study
AKY2398-1	Y8205 *lte1Δ::hphNT1* pTH17 *slk19Δ::natNT2*	This Study
AKY2435-1	Y8205 *lte1Δ::hphNT1* pTH17 *spo12Δ::natNT2 bfa1Δ::kanMX6*	This Study
AKY2436-1	Y8205 *lte1Δ::hphNT1* pTH17 *slk19Δ::natNT2 bfa1Δ::kanMX6*	This Study
AKY2437-1	Y8205 *lte1Δ::hphNT1* pTH17 *spo12Δ::natNT1 kin4Δ::kanMX6*	This Study
AKY2438-1	Y8205 lte1Δ::hphNT1 pTH17 *slk19Δ::natNT2 kin4Δ::kanMX6*	This Study
AKY2450	Y8205 *kin4Δ::URA3 lte1Δ::hphNT1*	This Study
AKY2461	Y8205 *kin4Δ::URA3 spo12Δ::hphNT1*	This Study
AKY2451	Y8205 *kin4Δ::URA3 lte1Δ::hphNT1 spo12Δ::natNT2*	This Study

ESM356-1	*MATa ura3-52 leu2Δ1 his3Δ200 trp1Δ63*	[59]
ESM356-9	*MATa ura3-52 leu2Δ1 his3Δ200*	Gift from Elmar Schiebel
ESM356-5	*MATa ura3-52 his3Δ200*	[20]
YPH499	*MATa ura3-52 lys2-801 ade2-101 trp1Δ63 his3Δ200 leu2Δ1*	[60]
YPH500	*MATalpha ura3-52 lys2-801 ade2-101 trp1Δ63 his3Δ200 leu2Δ1*	[60]
Y8205	*MATalpha can1Δ::*STE2pr-*SpHIS5 lyp1Δ::*STE3pr-*LEU2 his3Δ11 leu2Δ0 ura3Δ0*	[61]
K699	W303 *MATa can1-100 leu2-3,112 his3-11,15 trp1-1 ura3 ade2-1 GAL psi+*	[62]
RJD2862	W303 *MATa can1-100 leu2-3 his3-11 trp1-1 ura3-1 ade2-1 GAL psi+ net1Δ::his5 net1-6Cdk-TEV-9myc::TRP1*	[38]
SP4659	W303 *MATa ade2-1 trp1-1 leu2-3,112 his3-11,15 ura3 ssd1 bub2::BUB2R85A-URA3*	[63]
DOM0030	W303 *MATa ura3-1 trp1-1 leu2-3,112 his3-11 ade2-1 can1-100 GAL+ bar1Δ::hisG cdc28Δ::cdc28-as1*	[64]
SPY370-1	DOM0030 2µm-pGal-*CLB2-TAP-URA3*	This Study

Supplementary Table 2. Table of plasmids

Yeast strains	***Genotype***	***Source/Reference****
pRS315	CEN, *LEU2*	[60]
pRS316	CEN, *URA3*	[60]
pRS425	2µ, *LEU2*	[65]
yEP13	2µ, *LEU2*	[66]
YEplac181	2µ, *LEU2*	[67]
Yiplac128	Integration, *LEU2*	[67]
p416	*CEN, URA3*	[68]
pRS406	Integration, *URA3*	[69]
pRS306	Integration, *URA3*	[60]
pRS305	Integration, *LEU2*	[60]
pRS304	Integration, *TRP1*	[60]
pRS402	Integration, *ADE2*	[69]
pGW399	pRS316-*KAR9*	[31]
pNHK36	Ylp-*URA3*-GaL-*OsTIR1*-9Myc	[70]
pAKC104-1	pRS406-Gal1-*BFA1*	This Study
pGW412	p416-Gal1-*KIN4*	Gift from Elmar Schiebel
pGW410-1	pRS406-Gal1-*KIN4*	Gift from Elmar Schiebel
pAKC101	*pRS316-SPO12*	This Study
pTH20	yEP13-*SPO12*	Gift from Elmar Schiebel
pAKC115	pRS315-*SPO12*	This Study
pAK010	pRS305-*mcherry-TUB1*	[39]
pBK067	pRS304-*mCherry-TUB1*	[26]
pSM1027-1	pRS402-*GFP-TUB1*	[31]
pHM224	pRS305-*BFA1*	[27]
pAKC259-1	pRS305-$bfa1^{S274D,T288D,T340D, S454D, T465D, T500D}$ (*bfa1-6D*)	This Study
pAKC260-1	pRS305-$bfa1^{S274A, T288A, S454A, T340A, T500A, T465A}$ (*bfa1-6A*)	This Study
pRD41	pRS305-$bfa1^{T288A, S454A, T340A, T500A}$ (*bfa1-4A*)	This Study
p175	Ylplac128-*LTE1-GFP* (pMG58)	[71]
pAKC263	Ylplac128-$lte1^{K1273E}$-*GFP*	This Study
pAKC264	Ylplac128-$lte1^{F1387E}$-*GFP*	This Study
pTH17	pRS316-*LTE1*	[72]
pSM903-4	pRS316-*LTE1*	[72]
pSM912	pRS316-*DBF2*	Gift from Elmar Scheibel
pSM926	pRS316-*MOB1*	Gift from Elmar Scheibel
pKA59	pRS316-*TEM1*	Gift from Elmar Scheibel

pUG120	pRS316-*CDC14*	Gift from Elmar Scheibel
pCL33	pRS316-*CDC5*	Gift from Elmar Scheibel
pBS9	pRS316-*CDC15*	Gift from Elmar Scheibel
pMF642-2	pRS315-*ste20*^{K649R}	This Study
pTH35	pRS315-*STE20*	[72]
p7.2B	yEP13-TEM1	This Study
pWS68	YEplac181-CDC15	Gift from Elmar Scheibel
pILM34	pRS425-CDC14	This Study
pSM933	pRS425-DBF2	Gift from Elmar Scheibel
pGW070	pRS425-CDC5	Gift from Elmar Scheibel
pSM929	pRS425-MOB1	Gift from Elmar Scheibel
pR425-*TPK*1	pR425-*TPK*1	Gift from Elmar Scheibel
pAKC261-1	pMAL-c2x-BFA1	This Study
pAKC262-1	pMAL-c2x-*bfa1*$^{S274A, T288A, S454A, T340A, T500A, T465A}$	This Study

SUPPLEMENTARY METHODS

Yeast strains and plasmids

All strains and plasmids used in this study are listed in the Supplementary Tables 1 and 2. Yeast strains are isogenic with S288C unless otherwise specified. PCR-based methods were used for gene deletions and epitope tagging [73,74]. Genes of interest were expressed from their endogenous promoter unless otherwise stated. *GFP-TUB1*, *mCherry-TUB1,* and Gal1-*OsTIR*-9myc were integrated into the genome using integration plasmids [12,39,70,75]. Plasmids bearing wild type or mutated *BFA1* were integrated under endogenous *BFA1* promoter at the native *bfa1*Δ locus [27]. Plasmids bearing wild type or mutated *LTE1-GFP* were integrated in the *leu2*Δ*1* locus in *lte1*Δ strains [71]. *mob1*, *cdc15*, *bub2* and *net1* mutant genes were also integrated into the chromosomal DNA of strains that lack the corresponding wild type gene [38,58,63]. Most of the strains with *KAR9* deletion were maintained by complementation with *KAR9* on a centromeric *URA3*-based plasmid (pGW399) and analyzed for phenotypes shortly after the induction of plasmid loss by 5FOA selection [31].

Growth conditions

Yeast strains were grown in yeast extract peptone medium containing glucose and extra 0.1 mg/l adenine (YPAD) [76] at 30°C unless otherwise specified. For live-cell imaging, yeast cultures were grown in filter sterilised synthetic complete (SC) medium. Strains carrying plasmids were grown in SC medium lacking the corresponding amino acid. For induction of the Gal1 promoter yeast extract peptone medium containing raffinose (3%) and galactose (2%) (YP-Raf/Gal) was used. The curing of *URA3*-based plasmids from yeast strains was performed on agar plates containing 5-fluoro-orotic acid (5FOA, 1 mg/ml). Cells were synchronized in G1-phase by adding 10 µg/ml synthetic alpha-factor (Sigma, St. Louis, MO) to the logarithmically growing (log-phase) culture and subsequent further incubation of the culture until >90% of cells had formed mating projections. For nocodazole treatment, 15 µg/ml nocodazole (Sigma) was added to the culture media.

Protein methods

Yeast total protein extracts were obtained by cell lysis using NaOH and protein precipitation with Trichloroacetic acid [77]. Western blotting of SDS-PAGE gels was

performed using a semidry blotter [77]. Coomassie Brilliant Blue G-250 was used to stain SDS-PAGE gels. Antibodies were mouse anti-GFP (1:800, Roche: 11814460001, Basel, Switzerland), mouse anti-Tubulin (1:1000, Sigma: T6199) mouse anti-HA (12CA5, 1:1000, Sigma: SAB1305536), rabbit anti-Clb2 [27] (1:4000) and guinea pig anti-Sic1 [27] (1:1000), rabbit anti-Spc72 (against Spc72-N terminus [27], 1:150) and rabbit anti-Nud1 [27] (1:100). Secondary antibodies were goat anti-mouse (1:5000, Jackson ImmunoResearch Laboratories: 115-035-003, West Grove, PA), goat anti-rabbit (1:5000, Jackson ImmunoResearch Laboratories: 111-035-003) and goat anti-guinea pig (1:5000, Jackson ImmunoResearch Laboratories: 106-035-003) IgGs coupled to horseradish peroxidase.

MBP-Bfa1 and MBP-Bfa1-6A expression was induced in *E. coli* after 3h induction using IPTG (0.3 mM) at 30°C [27,78], and purified from cell lysates by affinity chromatography using amylose resin (New England Biolabs, Ipswich, MA). MBP-Bfa1 and MBP-Bfa1-6A were kept bound to the beads. Cdc28-as/Clb2-TAP complex was purified from *cdc28-as* yeast strains expressing Gal-*CLB2-TAP* on a 2µm plasmid [64]. Kinase complex was purified using TAP-purification protocol [79]. GST-Cdc5 (delta N70, S165D, T238D) and GST-Cdc5-KD (delta N70, N209A) were purified after induction of protein expression for 7 h using 1% Galactose [32].

For mass spectroscopy analysis, Bfa1-hbh [80] was purified from cells arrested in metaphase (Gal1-*CDC20* depletion) under denaturing conditions [81]. Briefly, 1000 ml of culture (2 x 10^7 cells/ml) pellet was lysed in a FastPrep FP120 Cell Disturber (MP Biomedicals, Santa Ana, CA) using acid-washed glass beads (Sigma-Aldrich). Lysis buffer contained 8 M urea, 300 mM NaCl, 0.5% Nonidet P-40, 50 mM sodium phosphate, 50 mM sodium phosphate buffer pH 8, 350 µg/ml benzamidine, 100 mM β-glycerophophate, 50 mM NaF, 5 mM $NaVO_3$, and complete EDTA-free protease inhibitor cocktail (Roche). Lysate was clarified by centrifugation at 3500 rpm 5 min. Total protein extract was diluted to 15 mg/ml and incubated with Ni-NTA sepharose bead slurry (GE Healthcare, Chicago, Illinois) for 6 h at room temperature. Proteins were eluted from the beads and incubated with Streptavidin beads [81]. After washing of the beads, proteins were eluted by incubating in Laemmli buffer supplemented with 4

μM of biotin for 10 min at 95 °C. The eluted fraction was loaded on SDS-PAGE and sent for MS analysis.

Uncut versions of all immunoblots shown in the figures are presented in supplementary information (Supplementary Fig. 12). Immunoblots shown are one representative out of two independent experiments.

Genome-wide synthetic lethality screens

Screens were performed in a 1536-colony format using a ROTOR colony pinning robot (Singer Instruments, Somerset, UK), with four technical replicates of each cross grouped 2x2 next to each other on the same plate. Screening was performed using the heterozygous diploid yeast deletion collection [82,83], where each strain carries a deletion of a single non-essential gene. The deletion collection was sporulated and mated with each of the query strains: AKY2450 (*kin4*Δ *lte1*Δ), AKY2461 (*kin4*Δ *spo12*Δ) and AKY2451 (*kin4*Δ *lte1*Δ *spo12*Δ). The resulting diploids were sporulated, followed by selection of haploids carrying simultaneously the query mutations and a gene deletion from the deletion collection [82,83]. Plates with the resulting haploid colonies were photographed. Colony sizes, normalized to the median of each plate, were determined from the photographs using SGATools [84]. For each cross, an average colony size was calculated from the four technical replicates. To identify gene deletions (deletion of a gene of interest, *goi*Δ) that are synthetic sick/lethal with the *kin4*Δ *lte1*Δ *spo12*Δ mutant, we calculated a delta score (D-score) as the difference between the colony size of each *kin4*Δ *lte1*Δ *spo12*Δ *goi*Δ cross and the minimum colony size of the two control crosses (*kin4*Δ *lte1*Δ *goi*Δ and *kin4*Δ *spo12*Δ *goi*Δ). Gene deletions synthetic sick/lethal with *kin4*Δ *lte1*Δ *spo12*Δ were then defined based on two criteria: D-score < -0.25 and *kin4*Δ *lte1*Δ *spo12*Δ *goi*Δ colony size < 0.75 (Supplementary Data 1).

SUPPLEMENTARY REFERENCES

1 Johnston, L. H., Eberly, S. L., Chapman, J. W., Araki, H. & Sugino, A. The product of the Saccharomyces cerevisiae cell cycle gene DBF2 has homology with protein kinases and is periodically expressed in the cell cycle. *Mol Cell Biol* **10**, 1358-1366 (1990).

2 Kitada, K., Johnson, A. L., Johnston, L. H. & Sugino, A. A multicopy suppressor gene of the Saccharomyces cerevisiae G1 cell cycle mutant gene dbf4 encodes a protein kinase and is identified as CDC5. *Mol Cell Biol* **13**, 4445-4457 (1993).

3 Schweitzer, B. & Philippsen, P. CDC15, an essential cell cycle gene in Saccharomyces cerevisiae, encodes a protein kinase domain. *Yeast* **7**, 265-273, doi:10.1002/yea.320070308 (1991).

4 Toyn, J. H., Araki, H., Sugino, A. & Johnston, L. H. The cell-cycle-regulated budding yeast gene DBF2, encoding a putative protein kinase, has a homologue that is not under cell-cycle control. *Gene* **104**, 63-70 (1991).

5 Wan, J., Xu, H. & Grunstein, M. CDC14 of Saccharomyces cerevisiae. Cloning, sequence analysis, and transcription during the cell cycle. *J Biol Chem* **267**, 11274-11280 (1992).

6 Shirayama, M., Matsui, Y. & Toh, E. A. The yeast TEM1 gene, which encodes a GTP-binding protein, is involved in termination of M phase. *Mol Cell Biol* **14**, 7476-7482 (1994).

7 Jaspersen, S. L., Charles, J. F., Tinker-Kulberg, R. L. & Morgan, D. O. A late mitotic regulatory network controlling cyclin destruction in Saccharomyces cerevisiae. *Mol Biol Cell* **9**, 2803-2817 (1998).

8 Parkes, V. & Johnston, L. H. SPO12 and SIT4 suppress mutations in DBF2, which encodes a cell cycle protein kinase that is periodically expressed. *Nucleic acids research* **20**, 5617-5623 (1992).

9 Toyn, J. H. & Johnston, L. H. Spo12 is a limiting factor that interacts with the cell cycle protein kinases Dbf2 and Dbf20, which are involved in mitotic chromatid disjunction. *Genetics* **135**, 963-971 (1993).

10 Shirayama, M., Matsui, Y. & Toh-e, A. Dominant mutant alleles of yeast protein kinase gene CDC15 suppress the lte1 defect in termination of M phase and genetically interact with CDC14. *Molecular & general genetics : MGG* **251**, 176-185 (1996).

11 Lee, S. E., Frenz, L. M., Wells, N. J., Johnson, A. L. & Johnston, L. H. Order of function of the budding-yeast mitotic exit-network proteins Tem1, Cdc15, Mob1, Dbf2, and Cdc5. *Curr Biol* **11**, 784-788, doi:S0960-9822(01)00228-7 [pii] (2001).

12 Shou, W. *et al.* Exit from mitosis is triggered by Tem1-dependent release of the protein phosphatase Cdc14 from nucleolar RENT complex. *Cell* **97**, 233-244, doi:S0092-8674(00)80733-3 [pii] (1999).

13 Toyn, J. H. & Johnston, L. H. The Dbf2 and Dbf20 protein kinases of budding yeast are activated after the metaphase to anaphase cell cycle transition. *EMBO J* **13**, 1103-1113 (1994).

14 Mah, A. S., Jang, J. & Deshaies, R. J. Protein kinase Cdc15 activates the Dbf2-Mob1 kinase complex. *Proc Natl Acad Sci U S A* **98**, 7325-7330, doi:10.1073/pnas.141098998
141098998 [pii] (2001).

15 Luca, F. C. *et al.* Saccharomyces cerevisiae Mob1p is required for cytokinesis and mitotic exit. *Mol Cell Biol* **21**, 6972-6983, doi:10.1128/MCB.21.20.6972-6983.2001 (2001).

16 Asakawa, K., Yoshida, S., Otake, F. & Toh-e, A. A novel functional domain of Cdc15 kinase is required for its interaction with Tem1 GTPase in Saccharomyces cerevisiae. *Genetics* **157**, 1437-1450 (2001).

17 Valerio-Santiago, M. & Monje-Casas, F. Tem1 localization to the spindle pole bodies is essential for mitotic exit and impairs spindle checkpoint function. *J Cell Biol* **192**, 599-614, doi:jcb.201007044 [pii]
10.1083/jcb.201007044 (2011).

18 Scarfone, I. & Piatti, S. Coupling spindle position with mitotic exit in budding yeast: The multifaceted role of the small GTPase Tem1. *Small GTPases* **6**, 1-6, doi:10.1080/21541248.2015.1109023 (2015).

19 Falk, J. E., Chan, L. Y. & Amon, A. Lte1 promotes mitotic exit by controlling the localization of the spindle position checkpoint kinase Kin4. *Proc Natl Acad Sci U S A* **108**, 12584-12590, doi:1107784108 [pii]
10.1073/pnas.1107784108 (2011).

20 Bertazzi, D. T., Kurtulmus, B. & Pereira, G. The cortical protein Lte1 promotes mitotic exit by inhibiting the spindle position checkpoint kinase Kin4. *J Cell Biol* **193**, 1033-1048, doi:jcb.201101056 [pii]
10.1083/jcb.201101056 (2011).

21 D'Aquino, K. E. *et al.* The protein kinase Kin4 inhibits exit from mitosis in response to spindle position defects. *Mol Cell* **19**, 223-234, doi:S1097-2765(05)01380-8 [pii]
10.1016/j.molcel.2005.06.005 (2005).

22 Pereira, G. & Schiebel, E. Kin4 kinase delays mitotic exit in response to spindle alignment defects. *Mol Cell* **19**, 209-221, doi:S1097-2765(05)01389-4 [pii]
10.1016/j.molcel.2005.05.030 (2005).

23 Bardin, A. J., Visintin, R. & Amon, A. A mechanism for coupling exit from mitosis to partitioning of the nucleus. *Cell* **102**, 21-31, doi:S0092-8674(00)00007-6 [pii] (2000).

24 Pereira, G., Hofken, T., Grindlay, J., Manson, C. & Schiebel, E. The Bub2p spindle checkpoint links nuclear migration with mitotic exit. *Mol Cell* **6**, 1-10, doi:S1097-2765(05)00017-1 [pii] (2000).

25 Adames, N. R., Oberle, J. R. & Cooper, J. A. The surveillance mechanism of the spindle position checkpoint in yeast. *J Cell Biol* **153**, 159-168 (2001).

26 Caydasi, A. K., Micoogullari, Y., Kurtulmus, B., Palani, S. & Pereira, G. The 14-3-3 protein Bmh1 functions in the spindle position checkpoint by breaking Bfa1 asymmetry at yeast centrosomes. *Mol Biol Cell* **25**, 2143-2151, doi:10.1091/mbc.E14-04-0890 (2014).

27 Maekawa, H., Priest, C., Lechner, J., Pereira, G. & Schiebel, E. The yeast centrosome translates the positional information of the anaphase spindle into a cell cycle signal. *J Cell Biol* **179**, 423-436, doi:10.1083/jcb.200705197 (2007).

28 Caydasi, A. K. & Pereira, G. Spindle alignment regulates the dynamic association of checkpoint proteins with yeast spindle pole bodies. *Dev Cell* **16**, 146-156, doi:S1534-5807(08)00440-1 [pii]
10.1016/j.devcel.2008.10.013 (2009).

29 Gryaznova, Y., Caydasi, A. K., Malengo, G., Sourjik, V. & Pereira, G. A FRET-based study reveals site-specific regulation of spindle position checkpoint proteins at yeast centrosomes. *Elife* **5**, doi:10.7554/eLife.14029 (2016).

30 Chan, L. Y. & Amon, A. The protein phosphatase 2A functions in the spindle position checkpoint by regulating the checkpoint kinase Kin4. *Genes Dev* **23**, 1639-1649 (2009).

31 Caydasi, A. K. *et al.* Elm1 kinase activates the spindle position checkpoint kinase Kin4. *J Cell Biol* **190**, 975-989, doi:jcb.201006151 [pii]
10.1083/jcb.201006151 (2010).

32 Geymonat, M., Spanos, A., Walker, P. A., Johnston, L. H. & Sedgwick, S. G. In vitro regulation of budding yeast Bfa1/Bub2 GAP activity by Cdc5. *J Biol Chem* **278**, 14591-14594, doi:10.1074/jbc.C300059200
C300059200 [pii] (2003).

33 Hu, F. *et al.* Regulation of the Bub2/Bfa1 GAP complex by Cdc5 and cell cycle checkpoints. *Cell* **107**, 655-665 (2001).

34 Visintin, R., Stegmeier, F. & Amon, A. The role of the polo kinase Cdc5 in controlling Cdc14 localization. *Mol Biol Cell* **14**, 4486-4498, doi:10.1091/mbc.E03-02-0095
E03-02-0095 [pii] (2003).
35 Queralt, E., Lehane, C., Novak, B. & Uhlmann, F. Downregulation of PP2A(Cdc55) phosphatase by separase initiates mitotic exit in budding yeast. *Cell* **125**, 719-732, doi:S0092-8674(06)00502-2 [pii]
10.1016/j.cell.2006.03.038 (2006).
36 Stegmeier, F., Visintin, R. & Amon, A. Separase, polo kinase, the kinetochore protein Slk19, and Spo12 function in a network that controls Cdc14 localization during early anaphase. *Cell* **108**, 207-220, doi:S0092867402006189 [pii] (2002).
37 Yoshida, S. & Toh-e, A. Budding yeast Cdc5 phosphorylates Net1 and assists Cdc14 release from the nucleolus. *Biochem Biophys Res Commun* **294**, 687-691, doi:10.1016/S0006-291X(02)00544-2
S0006-291X(02)00544-2 [pii] (2002).
38 Azzam, R. *et al.* Phosphorylation by cyclin B-Cdk underlies release of mitotic exit activator Cdc14 from the nucleolus. *Science* **305**, 516-519, doi:10.1126/science.1099402 (2004).
39 Khmelinskii, A., Lawrence, C., Roostalu, J. & Schiebel, E. Cdc14-regulated midzone assembly controls anaphase B. *J Cell Biol* **177**, 981-993, doi:jcb.200702145 [pii]
10.1083/jcb.200702145 (2007).
40 Khmelinskii, A., Roostalu, J., Roque, H., Antony, C. & Schiebel, E. Phosphorylation-dependent protein interactions at the spindle midzone mediate cell cycle regulation of spindle elongation. *Dev Cell* **17**, 244-256, doi:S1534-5807(09)00252-4 [pii]
10.1016/j.devcel.2009.06.011 (2009).
41 Ross, K. E. & Cohen-Fix, O. A role for the FEAR pathway in nuclear positioning during anaphase. *Dev Cell* **6**, 729-735, doi:S1534580704001285 [pii] (2004).
42 Rock, J. M. & Amon, A. The FEAR network. *Curr Biol* **19**, R1063-1068, doi:S0960-9822(09)01777-1 [pii]
10.1016/j.cub.2009.10.002 (2009).
43 Sullivan, M., Higuchi, T., Katis, V. L. & Uhlmann, F. Cdc14 phosphatase induces rDNA condensation and resolves cohesin-independent cohesion during budding yeast anaphase. *Cell* **117**, 471-482, doi:S0092867404004155 [pii] (2004).
44 D'Amours, D., Stegmeier, F. & Amon, A. Cdc14 and condensin control the dissolution of cohesin-independent chromosome linkages at repeated DNA. *Cell* **117**, 455-469, doi:S0092867404004131 [pii] (2004).
45 Torres-Rosell, J., Machin, F., Jarmuz, A. & Aragon, L. Nucleolar segregation lags behind the rest of the genome and requires Cdc14p activation by the FEAR network. *Cell Cycle* **3**, 496-502 (2004).
46 Wang, B. D., Yong-Gonzalez, V. & Strunnikov, A. V. Cdc14p/FEAR pathway controls segregation of nucleolus in S. cerevisiae by facilitating condensin targeting to rDNA chromatin in anaphase. *Cell Cycle* **3**, 960-967 (2004).
47 Higuchi, T. & Uhlmann, F. Stabilization of microtubule dynamics at anaphase onset promotes chromosome segregation. *Nature* **433**, 171-176, doi:10.1038/nature03240 (2005).
48 Pereira, G. & Schiebel, E. Cdc14 phosphatase resolves the rDNA segregation delay. *Nat Cell Biol* **6**, 473-475, doi:10.1038/ncb0604-473
ncb0604-473 [pii] (2004).
49 Roccuzzo, M., Visintin, C., Tili, F. & Visintin, R. FEAR-mediated activation of Cdc14 is the limiting step for spindle elongation and anaphase progression. *Nat Cell Biol* **17**, 251-261, doi:10.1038/ncb3105 (2015).
50 Woodbury, E. L. & Morgan, D. O. Cdk and APC activities limit the spindle-stabilizing function of Fin1 to anaphase. *Nat Cell Biol* **9**, 106-112, doi:10.1038/ncb1523 (2007).

51 Tomson, B. N. *et al.* Regulation of Spo12 phosphorylation and its essential role in the FEAR network. *Curr Biol* **19**, 449-460, doi:10.1016/j.cub.2009.02.024 (2009).
52 Rossio, V. & Yoshida, S. Spatial regulation of Cdc55-PP2A by Zds1/Zds2 controls mitotic entry and mitotic exit in budding yeast. *J Cell Biol* **193**, 445-454, doi:10.1083/jcb.201101134 (2011).
53 Queralt, E. & Uhlmann, F. Separase cooperates with Zds1 and Zds2 to activate Cdc14 phosphatase in early anaphase. *J Cell Biol* **182**, 873-883, doi:10.1083/jcb.200801054 (2008).
54 Calabria, I., Baro, B., Rodriguez-Rodriguez, J. A., Russinol, N. & Queralt, E. Zds1 regulates PP2A(Cdc55) activity and Cdc14 activation during mitotic exit through its Zds_C motif. *J Cell Sci* **125**, 2875-2884, doi:10.1242/jcs.097865 (2012).
55 Stegmeier, F. *et al.* The replication fork block protein Fob1 functions as a negative regulator of the FEAR network. *Curr Biol* **14**, 467-480, doi:10.1016/j.cub.2004.03.009 (2004).
56 Pereira, G., Manson, C., Grindlay, J. & Schiebel, E. Regulation of the Bfa1p-Bub2p complex at spindle pole bodies by the cell cycle phosphatase Cdc14p. *J Cell Biol* **157**, 367-379, doi:10.1083/jcb.200112085
jcb.200112085 [pii] (2002).
57 Pereira, G. & Schiebel, E. Separase regulates INCENP-Aurora B anaphase spindle function through Cdc14. *Science* **302**, 2120-2124, doi:10.1126/science.1091936 (2003).
58 Konig, C., Maekawa, H. & Schiebel, E. Mutual regulation of cyclin-dependent kinase and the mitotic exit network. *J Cell Biol* **188**, 351-368, doi:jcb.200911128 [pii]
10.1083/jcb.200911128 (2010).
59 Pereira, G., Tanaka, T. U., Nasmyth, K. & Schiebel, E. Modes of spindle pole body inheritance and segregation of the Bfa1p-Bub2p checkpoint protein complex. *EMBO J* **20**, 6359-6370, doi:10.1093/emboj/20.22.6359 (2001).
60 Sikorski, R. S. & Hieter, P. A system of shuttle vectors and yeast host strains designed for efficient manipulation of DNA in Saccharomyces cerevisiae. *Genetics* **122**, 19-27 (1989).
61 Tong, A. H. & Boone, C. Synthetic genetic array analysis in Saccharomyces cerevisiae. *Methods Mol Biol* **313**, 171-192 (2006).
62 Nasmyth, K., Adolf, G., Lydall, D. & Seddon, A. The identification of a second cell cycle control on the HO promoter in yeast: cell cycle regulation of SW15 nuclear entry. *Cell* **62**, 631-647 (1990).
63 Fraschini, R., D'Ambrosio, C., Venturetti, M., Lucchini, G. & Piatti, S. Disappearance of the budding yeast Bub2-Bfa1 complex from the mother-bound spindle pole contributes to mitotic exit. *J Cell Biol* **172**, 335-346, doi:jcb.200507162 [pii]
10.1083/jcb.200507162 (2006).
64 Ubersax, J. A. *et al.* Targets of the cyclin-dependent kinase Cdk1. *Nature* **425**, 859-864, doi:10.1038/nature02062 (2003).
65 Christianson, T. W., Sikorski, R. S., Dante, M., Shero, J. H. & Hieter, P. Multifunctional yeast high-copy-number shuttle vectors. *Gene* **110**, 119-122 (1992).
66 Broach, J. R., Strathern, J. N. & Hicks, J. B. Transformation in yeast: development of a hybrid cloning vector and isolation of the CAN1 gene. *Gene* **8**, 121-133 (1979).
67 Gietz, R. D. & Sugino, A. New yeast-Escherichia coli shuttle vectors constructed with in vitro mutagenized yeast genes lacking six-base pair restriction sites. *Gene* **74**, 527-534 (1988).
68 Mumberg, D., Muller, R. & Funk, M. Yeast vectors for the controlled expression of heterologous proteins in different genetic backgrounds. *Gene* **156**, 119-122 (1995).
69 Brachmann, C. B. *et al.* Designer deletion strains derived from Saccharomyces cerevisiae S288C: a useful set of strains and plasmids for PCR-mediated gene disruption and other applications. *Yeast* **14**, 115-132, doi:10.1002/(SICI)1097-0061(19980130)14:2<115::AID-YEA204>3.0.CO;2-2 (1998).

70 Nishimura, K., Fukagawa, T., Takisawa, H., Kakimoto, T. & Kanemaki, M. An auxin-based degron system for the rapid depletion of proteins in nonplant cells. *Nat Methods* **6**, 917-922, doi:10.1038/nmeth.1401 (2009).
71 Geymonat, M., Spanos, A., de Bettignies, G. & Sedgwick, S. G. Lte1 contributes to Bfa1 localization rather than stimulating nucleotide exchange by Tem1. *J Cell Biol* **187**, 497-511, doi:jcb.200905114 [pii]
10.1083/jcb.200905114 (2009).
72 Hofken, T. & Schiebel, E. A role for cell polarity proteins in mitotic exit. *EMBO J* **21**, 4851-4862 (2002).
73 Janke, C. *et al.* A versatile toolbox for PCR-based tagging of yeast genes: new fluorescent proteins, more markers and promoter substitution cassettes. *Yeast* **21**, 947-962 (2004).
74 Knop, M. *et al.* Epitope tagging of yeast genes using a PCR-based strategy: more tags and improved practical routines. *Yeast* **15**, 963-972 (1999).
75 Straight, A. F., Marshall, W. F., Sedat, J. W. & Murray, A. W. Mitosis in living budding yeast: anaphase A but no metaphase plate. *Science* **277**, 574-578 (1997).
76 Sherman, F. Getting started with yeast. *Methods Enzymol* **194**, 3-21 (1991).
77 Meitinger, F., Palani, S. & Pereira, G. Detection of Phosphorylation Status of Cytokinetic Components. *Methods Mol Biol* **1369**, 219-237, doi:10.1007/978-1-4939-3145-3_16 (2016).
78 Geymonat, M., Spanos, A. & Sedgwick, S. Production of mitotic regulators using an autoselection system for protein expression in budding yeast. *Methods Mol Biol* **545**, 63-80 (2009).
79 Puig, O. *et al.* The tandem affinity purification (TAP) method: a general procedure of protein complex purification. *Methods* **24**, 218-229, doi:10.1006/meth.2001.1183 (2001).
80 Tagwerker, C. *et al.* HB tag modules for PCR-based gene tagging and tandem affinity purification in Saccharomyces cerevisiae. *Yeast* **23**, 623-632, doi:10.1002/yea.1380 (2006).
81 Tagwerker, C. *et al.* A tandem affinity tag for two-step purification under fully denaturing conditions: application in ubiquitin profiling and protein complex identification combined with in vivocross-linking. *Molecular & cellular proteomics : MCP* **5**, 737-748, doi:10.1074/mcp.M500368-MCP200 (2006).
82 Baryshnikova, A. *et al.* Synthetic genetic array (SGA) analysis in Saccharomyces cerevisiae and Schizosaccharomyces pombe. *Methods Enzymol* **470**, 145-179, doi:10.1016/S0076-6879(10)70007-0 (2010).
83 Winzeler, E. A. *et al.* Functional characterization of the S. cerevisiae genome by gene deletion and parallel analysis. *Science* **285**, 901-906 (1999).
84 Wagih, O. *et al.* SGAtools: one-stop analysis and visualization of array-based genetic interaction screens. *Nucleic acids research* **41**, W591-596, doi:10.1093/nar/gkt400 (2013).
